# Meaning-Focused Coping in University Students in Hong Kong During the COVID-19 Pandemic: A Qualitative Study

**DOI:** 10.3390/ijerph22040614

**Published:** 2025-04-15

**Authors:** Tingyin Wong, Daniel T. L. Shek

**Affiliations:** Department of Applied Social Sciences, The Hong Kong Polytechnic University, Hong Kong 999077, China; tingyin.wong@polyu.edu.hk

**Keywords:** meaning-focused coping, perceived benefits, COVID-19 pandemic, university students, thematic analysis

## Abstract

Many studies were conducted during and after the COVID-19 pandemic to examine its impact on young people’s well-being. However, most studies are quantitative ones focusing on the negative impact of the pandemic on young people. In contrast, there are few studies examining the benefits of the pandemic using a qualitative methodology. Hence, we conducted focus group interviews to understand meaning-focused coping in 56 university students from late December 2022 to mid-January 2023 when Hong Kong was approaching the end of the pandemic. Thematic analysis using a deductive analytical approach based on the Revised Stress and Coping Model was applied during data analysis. The results showed that most students were able to generate positive experiences and emotions during the pandemic. Many of them reported improved mental well-being during the pandemic by infusing ordinary events with meaning. They engaged in adaptive goal processes by having a more positive attitude towards life. Students also found benefits in terms of enhanced personal strengths, better interpersonal relationships, and improved pandemic-related abilities. Furthermore, the current study compared the difference in the use of meaning-focused coping and perceived benefits by students with family or personal financial difficulties, students who coped well, and Mainland Chinese students studying in Hong Kong. This paper provides an alternative picture of the impact of the COVID-19 pandemic on university students.

## 1. Introduction

While many studies have examined the negative impact of the COVID-19 pandemic on individuals using quantitative methods, relatively fewer studies have examined the positive impact of the pandemic on human functioning using a qualitative methodology. As such, the current study examined young people’s meaning-focused coping during the COVID-19 pandemic, which offers an innovative and alternative perspective to understand disaster-related coping.

Everyday stressors and natural disasters may incite individuals to generate new meaning, which could improve individuals’ adjustment, growth, and well-being [1,2]. During the COVID-19 pandemic, some studies were conducted to understand the use of meaning-focused coping [3,4,5]. For example, analyzing adults in 30 countries, Eisenbeck et al. [6] found that meaning-focused coping was a stronger predictor of individuals’ physical and mental health than emotion-focused and problem-focused coping during the initial stages of the pandemic. Examining American adults during the early lockdown period in April 2020, Park et al. [7] found that meaning-focused coping predicted improved adjustment and resilience. For university students, researchers have studied their meaning-focused coping and post-traumatic growth during the pandemic [8,9,10,11]. For example, August and Dapkewicz [12] examined college students living under the shelter-in-place orders during the pandemic and found that “benefit finding” was a commonly applied coping strategy. Furthermore, students with self-related benefits were less likely to report mental distress, including stress, fear, and anxiety.

Although there are some isolated quantitative studies on young people’s meaning-focused coping and post-traumatic growth from the pandemic, there are even fewer qualitative studies on related topics. The current study could fill in this knowledge gap by investigating Hong Kong university students’ meaning-focused coping and perceived benefits of the COVID-19 pandemic. This could also contribute to the meaning literature, as there are limited empirical research findings to test theories of meaning and meaning making [13]. Moreover, the study of meaning-focused coping would assist in the construction and reconstruction of meaning of the general population after the pandemic, which would benefit their mental health and holistic well-being in the long run [14].

## 2. Theoretical Framework

Scholars have investigated positive changes in individuals after experiencing trauma or adversity. These positive changes are named adversarial growth, post-traumatic growth, stress-related growth, thriving, or benefit finding [15]. Adversarial, post-traumatic, and stress-related growth refer to positive changes in individuals resulting from a stressful situation. Individuals may even achieve a higher level of functioning after the stressful experience in five areas: personal strength, spirituality, relationships with others, life appreciation, and new possibilities [16,17]. People who thrive may be less reactive to and have faster recovery from subsequent stressors. They may also consistently show a higher level of functioning [18]. For benefit finding and benefit reminding, Tennen and Affleck [19] suggested their relevance to positive psychology. Rather than “a form of denial or a maladaptive reality distortion” [19] (p. 589), benefit finding is assumed to be a selective appraisal and coping strategy, and it happens in later adjustment to adversity. However, coping is distinguished from adaptive behaviors because the former requires effort, beliefs, and strategies. Furthermore, benefit finding can only be considered a coping strategy if the discovery of benefits can be used to comfort the self during adversity. Similarly, Frankl [20] stressed that a person no longer suffers when he/she finds meaning in suffering. Additionally, a related concept, “psychological adjustment to stress”, is not clearly defined in the literature. Some empirical studies measured participants’ depressive symptoms, satisfaction with life, or positive states of mind to examine their psychological adjustment after a stressful experience [16,21], while the meta-analysis conducted by Ano and Vasconcelles [22] divided psychological adjustment into 18 positive outcomes (e.g., acceptance, stress-related growth) and 20 negative outcomes (e.g., negative affect, post-traumatic stress disorder symptoms). The current study contributes to the literature by qualitatively demonstrating young people’s meaning-focused coping and perceived benefits from the COVID-19 pandemic, which is closely related to the concept of psychological adjustment to stress.

The role of positive emotions in the stress process was first introduced by Lazarus et al. [23]. It was suggested that positive emotions could motivate coping (as “sustainers”), offer a brief relief from distress (as “breathers”), and supplement the coping resources (as “restorers”) of an individual. In the original Transactional Model of Stress and Coping proposed by Lazarus and Folkman [24], after the primary appraisal (i.e., personal significance of an event) and secondary appraisal (i.e., coping resources) of a stressful situation, there are two major coping functions or purposes. They are regulation of distress when the stressful problem cannot be resolved (i.e., emotion-focused coping) and management or solving of the problem (i.e., problem-focused coping). However, when the outcome is unfavorable, individuals may enter the reappraisal stage. Positive reappraisal of meaning enables individuals to have personal growth and perceive benefits during the stress process, which could lead to positive emotions [25]. Positive emotions could restore function and coping resources in a person’s physiological, psychological, and social dimensions [26]. The Revised Stress and Coping Model was proposed by Folkman [25] and further advanced by Folkman [26].

**Figure 1:**
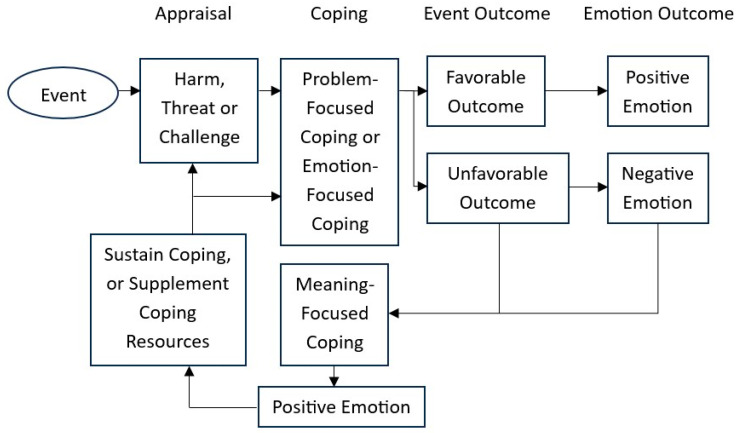
Revised Stress and Coping Model (adapted from Folkman [26]).

Meaning-focused coping is appraisal-based coping in which individuals sustain coping and maintain well-being during stressful situations by adjusting their beliefs, values, and goals [26]. Meaning-focused coping also involves the reappraisal of global and situation meaning to help an individual deal with stressful situations when there are no immediately available solutions to the problems [27]. It encompasses loss of original meaning, gain of new meaning, and complex emotional changes. According to Folkman [26], there are five types of meaning-focused coping strategies, which are benefit finding, benefit reminding, adaptive goal processes, reordering priorities, and infusing ordinary events with positive meaning. This theory forms a theoretical basis for the current study on developing codes related to positive meaning and benefits gained from the COVID-19 pandemic, which is graphically illustrated in Figure 1. This theory has been widely used by researchers before and after the outbreak of the pandemic to investigate how individuals derive positive meaning in relation to children and adolescent development [28,29], chronic diseases [30,31,32], and disasters [1,33,34,35]. According to Boyatzis [36], a theory-driven approach to qualitative research could extend, replicate, or rebut prior research findings. The current study contributes to the coping literature by testing and extending a prior theory on meaning-focused coping.

Researchers have highlighted the significant associations between meaning making, positive affect, and adjustment [13]. Positive emotions facilitate individuals’ ability to rebuild cognitive, physical, and social resources, which could benefit long-term adaptation to stressful situations [37]. Positive emotions could also reduce the negative psychological and physiological influence of stressful experiences [38,39]. The Broaden-and-Build Theory of Positive Emotions argues that positive emotions widen an individual’s momentary thought–action repertoire and provide resources for an individual to cope with or adjust to stressful situations [40,41]. A review of the literature on meaning by Park [13] also showed various empirical studies linking meaning making to positive and negative affect [42,43,44,45]. The current study extended this line of research and examined the positive reappraisal of meaning leading to the generation of positive emotions during adversity.

During the COVID-19 pandemic, researchers examined young people’s positive and negative affect and daily emotional well-being using the 20-item Positive and Negative Affect Schedule scale (PANAS) [46], in which “Positive Affect” refers to the feeling of being active, alert, and enthusiastic and “Negative Affect” refers to unpleasurable engagement or subjective distress. Deng et al. [47] found no significant change in negative affect but a significant decrease in positive affect in US adolescents during the pandemic, probably due to the restriction on social or sporting activities at the initial stages of the pandemic. Comparing the positive affect of higher education institution students in Mainland China before and during the pandemic, Wang et al. [48] found that these students had a lower level of positive affect during the pandemic [49]. In the current study, the use of a qualitative research approach enabled us to examine a broader range of positive emotions compared to the ten descriptors of positive affect in the PANAS scale.

Although qualitative studies conducted in the US [12] and Mainland China [10] have examined university students’ meaning-focused coping or perceived benefits during the COVID-19 pandemic, there is no related qualitative study on Hong Kong university students. In Hong Kong, quantitative studies conducted by Yeung et al. [34,35] showed that religious coping was associated with adversarial growth in Filipina domestic helpers, and more frequent emotional processing was related to adversarial growth in nurses amid the pandemic. Quantitative studies on Hong Kong university students also showed that positive psychological attributes (i.e., resilience, emotional competence, and positive beliefs related to adversity) and ecological protective factors (i.e., Chinese cultural beliefs of adversity) were positively associated with well-being during the pandemic [50,51,52,53]. Although Hong Kong is a place with strong traditional Chinese cultural influence, it is also subject to the influence of Western values. Hence, the current qualitative study could fill this knowledge gap and contribute to a more comprehensive understanding of meaning-focused coping and perceived benefits among young people in Hong Kong during the pandemic.

The objective of the current study was to qualitatively illustrate the use of meaning-focused coping and perceived benefits of Hong Kong university students during the COVID-19 pandemic. Based on the research objective, the following research questions were investigated:

1. What meaning-focused coping strategies did Hong Kong university students use during the COVID-19 pandemic?
2. Did Hong Kong university students experience post-traumatic or stress-related growth from the COVID-19 pandemic?

In the current study, the five categories of meaning-focused coping proposed by Folkman [26] guided the deductive analysis process, while sub-themes of meaning-focused coping were formed from the narratives of research participants. Moreover, according to Tennen and Affleck [19], benefit finding can be regarded as a coping strategy for individuals only if the discovery of benefits is used to comfort themselves during difficult times. As such, perceived benefits that were not directed towards the generation of positive affect were grouped into a new category, “perceived benefits”.

## 3. Materials and Methods

### 3.1. Research Design

The current study applied the guidelines on deductive qualitative research proposed by Fife and Gossner [54]. First, we developed the research questions and selected the guiding theory, which is the Revised Stress and Coping Model proposed by Folkman [26]. Second, we operationalized the theory by extracting the five categories of meaning-focused coping from the guiding theory. Third, we sampled research participants through purposive sampling to facilitate in-depth analysis of the theory. Fourth, we began coding the data by gathering the brief notes written during the interviews and transcribing the audio recordings of the focus group interviews. The interviews were transcribed through verbatim transcription and then translated from Chinese to English. The notes and transcriptions were coded by generating and categorizing keywords, codes, and themes based on the five categories of meaning-focused coping. The codes were revised several times during the coding process based on the authors’ regular discussions. Fifth, the evidence was interpreted, and a proposal to confirm or revise the guiding theory was suggested.

The current study applied thematic analysis during qualitative data analysis [55]. This was underpinned by ontological relativism and epistemological post-positivism, which state that reality is created by our mind and that knowledge construction is objective and developed based on previous theories and empirical evidence [56]. The analytical process in thematic analysis balances descriptive analysis, which captures data patterns or trends, and interpretive analysis, which analyzes the data subjectively [57], while post-positivist paradigms have a stronger emphasis on describing the objective reality. Thematic analysis is also applicable to the deductive approach to qualitative research because of its structured nature and applicability to theory testing [58]. The authors’ reflexivity statements are presented in Appendix A.1, which shows that authors’ subjectivity could serve as a resource in thematic analysis [55].

### 3.2. Sampling and Procedures

Research participants were sampled through purposeful sampling for focus group interviews from 29 December 2022 to 13 January 2023 [50,51]. To encourage students’ participation, financial incentives were offered. When the interviews were conducted, Hong Kong was beginning to return to normal. Some local universities had already returned to face-to-face teaching and learning as of September 2022. Starting 29 December 2022, mandatory nucleic acid testing and social distancing measures were canceled for people entering Hong Kong. Therefore, research participants had already undergone three years of the pandemic when the interviews were conducted, and they were beginning to return to normal life. Compared with other countries or regions, Hong Kong had a relatively long period of pandemic restriction imposed by the government’s preventive measures. Taking the United States as an example, mask wearing was no longer legally enforced in May 2022, and the order requiring passengers boarding a flight to the United States to have negative COVID-19 test results was canceled in June 2022 [59,60]. However, related policies in Hong Kong were canceled only in March 2023 and December 2022, respectively. In addition, there was a major shift in zero-COVID-19 policy in Mainland China in early December 2022. The shift in policy caused a national outbreak, which led to a significant impact on the economy and the social life of people in Mainland China [61] and may have influenced the coping and perceived benefits of Mainland Chinese students studying in Hong Kong at the time. The current study could contribute to the literature by examining the meaning-focused coping and perceived benefits of young people in Hong Kong at this exceptional timepoint.

For the demographic background of the research participants, they were all Year 2 to 4 undergraduate students aged 19 to 25 from one large-scale research university in Hong Kong. They gave consent to data collection and interview recording. In the consent form, research procedures, including voluntary participation, identity protection, and data storage security, were explained. To protect their personal identity, research participants were acknowledged using code numbers during the interviews. Moreover, participants were divided into different categories based on their responses to a short survey conducted before the interviews. Specifically, students who answered “yes” to the question “Did you or your family face financial difficulties during the pandemic?” were categorized as “students with financial difficulties”. Students who answered “yes” to the question “Despite the difficulties I have faced during the pandemic, I consider myself to have coped well. Do you agree with this statement?” were categorized as “students who coped well”. And students who answered “mainland student” to the question “Are you a local, mainland, or international student?” were categorized as “Mainland Chinese students studying in Hong Kong”. The current study focused on these three categories of Hong Kong university students. For each category, around 20 students were recruited, and they were conveniently divided into 3 to 4 focus groups, resulting in 56 participants in 10 focus groups. The three categories, related questions asked before the interviews, participant codes, and the number of participants in each category are listed in Table 1. In the participant codes, the first letter (F, C, and M) denotes students with financial difficulties, students who coped well, and Mainland Chinese students, respectively, the second letter (A, B, C, D) represents the different focus groups that the students were divided into, and the last number (1 to 9) denotes a random number assigned to students in the focus groups. Based on the criteria outlined by Malterud et al. [62], including theoretical framework application, high sample specificity, and high quality of qualitative data collected, the current sample size provides adequate information power.

The three categories (i.e., students with financial difficulties, students who coped well, and Mainland Chinese students) were chosen based on a review of the literature, showing the most representative challenges faced by Hong Kong university students amid the pandemic. Relatively few studies have investigated how university students coped with financial difficulties or challenges related to their international student status during the pandemic. The current study could fill this knowledge gap and extend the findings in the literature. For financial difficulties, a study in Hong Kong showed that unemployment, personal and family financial difficulties, and living alone were significantly associated with university students’ mental distress during the pandemic [51]. The current study used a qualitative approach to further explore the use of meaning-focused coping by university students with financial difficulties. Furthermore, Mainland Chinese students make up a significant percentage of university students in Hong Kong. One study in Hong Kong found that local students showed more severe depressive symptoms than international students because of the cumulative stresses of the 2019 social unrest, the COVID-19 pandemic, and the small living space [63]. This finding contradicts studies on international students in other place; for example, Lai et al. [64] showed that international students suffered from more severe mental distress if they stayed in the country where they attended university amid the pandemic. The current study could further explore international students’ meaning-focused coping during this special period of time. Moreover, resilience is defined as “an individual’s capacity for adapting to change and to stressful events in healthy and flexible ways” [65] (p. 102) based on the positive youth development constructs proposed by Catalano et al. [65], so students who coped well with the stressors of the pandemic could be regarded as resilient students. Studies have highlighted the significant relationship between resistance and mental well-being during the pandemic. A study on Hong Kong university students revealed that students with high resilience had better support systems and less family distress, and they were less likely to have anxiety and depressive symptoms [66]. By comparing resilient students with students facing financial difficulties and Mainland Chinese students, we could gain a better understanding of whether these factors could affect the use of meaning-focused coping and perceived benefits in young people during the pandemic.

Focus group interviews were conducted via Zoom because of the social distancing measures in Hong Kong at the time. The moderators of each interview consisted of an experienced researcher and a research assistant. A semi-structured interview guide assisted the moderators in conducting the interviews (Appendix A.2). After the moderators asked each interview question, research participants took turns answering the question. Both moderators asked follow-up questions. Brief notes were recorded by the research assistants during the interviews. The interviews were audio-recorded and transcribed for later analysis.

### 3.3. Data Analysis

The current study employed a thematic analytic method to analyze the focus group interview data, which is commonly applied in the fields of social science and psychology. This methodology centers around the research questions to search for themes or patterns in qualitative data [67]. Thematic analysis is divided into “coding reliability”, “codebook”, and “reflexive” approaches [55]. The “codebook” approach, which is guided by a pre-determined codebook or coding frame, was applied in the current study. This approach sits between more structured coding (small q) and an organic or flexible approach (Big Q) [68]. This approach adopts both the philosophy of the “reflexive” approach and the structured approach to coding, and it includes the use of multiple coders and coding reliability measures to test the reliability of the analysis [69]. Furthermore, the current study applied the deductive analytical approach, which examines pre-existing theories through empirical data and facilitates the testing and refinement of theories [54,70]. This enabled our findings to be built upon other studies and facilitated the development of a body of knowledge in the field [71]. The current study could contribute to testing and refining the five strategies of meaning-focused coping, which is the theoretical foundation of the meaning-focused coping concept.

### 3.4. Rigor and Trustworthiness

The current study was guided by the qualitative research principles stated by Shek et al. [72]. Key principles include asserting the study’s philosophical base, explaining the choice of sample size, describing the process of data collection, justifying the researchers’ subjectivity and potential biases through their reflexivity statements, testing inter-rater reliability to ensure analytical reliability, maintaining the study’s high auditability through detailed explanation of the researchers’ decision trail and offering more illustrative quotes of the research participants, checking negative cases and alternative explanation of the findings, and detailing the study’s limitation. Furthermore, the current study maintained methodological integrity by selecting procedures that were conducive to producing insightful findings to answer the research questions and by maintaining consistency with the tradition of inquiry and the phenomenon under study [73].

## 4. Results

Out of the five types of meaning-focused coping strategies proposed by Folkman [26], a significant number of students applied the “benefit finding” strategy (*n* = 35, 63%), “infusing ordinary events with meaning” (*n* = 23, 41%), and “realigning priorities” (*n* = 19, 34%), with only a few students employing “adaptive goal processes” (*n* = 9, 16%) and “benefit reminding” (*n* = 2, 3.6%). The current study also found that students perceived benefits related to their improved interpersonal relationships, enhanced personal strengths, and strengthened skills specific to the pandemic, which may not be related to the generation of positive emotions during adversity, so related codes were grouped under a new theme, “perceived benefits” (*n* = 30, 54%). To ensure the reliability of the analysis, an inter-rater reliability test was conducted. We randomly selected 20 quotations from the focus group interviews, and they were presented to two independent researchers to re-code the quotations. The inter-rater reliability test results showed satisfactory reliability of our analysis (percentage agreement on presence ≥ 70%) [36]. Table 2 summarizes the themes and sub-themes, while Table A1 in Appendix A.2 lists the use of meaning-focused coping and perceived benefits by each participant. One participant may use multiple coping strategies, so the frequencies were calculated by subtracting multiple uses across sub-themes. For additional illustrative quotes in each category, please see Supplemental Table S1.

### 4.1. Benefit Finding

“Benefit finding” comprises the positive effects that result from a traumatic event, which is a form of coping that reduces mental distress in relation to a continuing challenge. According to Tennen and Affleck [19], benefit finding can only be considered a coping strategy if the discovery of benefits can be used to comfort the self during adversity. The current study found that students learned to be more accepting, flexible, and open-minded and worried less (*n* = 11, 20%), had an increased sense of responsibility and self-perceived personal growth (*n* = 4, 7.1%), cherished relationships with family members and friends (*n* = 19, 34%), and were more open to communicating with strangers and showing care to society (*n* = 8, 14%).

First, students learned to be more accepting, flexible, and open-minded and worried less because many things could not be controlled during the pandemic, as a student explained:

*I believe the pandemic has helped me to reflect on the meaning of life and strengthened my personal growth. Personally, I used to be someone who planned many things and worried about many things in the future. However, the pandemic has made me realized that many things can unfold naturally. It’s important to go with the flow and be adaptable because there are so many variables at play. No one would anticipate that people in Hong Kong would experience two years without travel and we would have to wear face masks for three years. No one could predict this situation beforehand. So, although I used to plan many things for the next two to three years, the reality is that the world is filled with uncertainty, and it’s not surprising for us to encounter another pandemic in the next few years. As a result, I’ve become more optimistic and worry less about the future.*

(Student who coped well, group B, C-B-3).

Furthermore, students with financial difficulties stated that their sense of responsibility increased because they had more duties during the pandemic, including purchasing food and anti-pandemic supplies for their families and being aware of the constantly changing preventive measures by the government, which led to self-perceived personal growth. Financial difficulties facing their families also made them realize the importance of making money to support their families in the future, which was regarded by students as a sense of becoming more mature, as a student stated:

*My sense of responsibility has improved a lot. As we have discussed, especially about purchasing things [for the family], I feel that I need to be responsible for some duties at home, and purchasing food is especially important. I feel that I have some responsibilities. Additionally, I have to work, so my sense of responsibility has increased a lot.*

(Student with financial difficulty, group A, F-A-2).

For interpersonal relationships, participants stated that they learned to cherish and appreciate their relationships with family members and friends. They learned to turn fear into mutual encouragement because of the challenges they experienced together during the pandemic. They also learned to be more tolerant and considerate towards their family members and friends. Furthermore, because of difficulties in meeting face to face with friends, students cherished every time they could meet their family members and friends. The pandemic also caused the death of many people, including participants’ family members and friends, which made them cherish their relationships even more, as a student stated:

*I think what I have learned is to cherish. Before the pandemic, making appointment with friends was a very ordinary thing. Going to different places or travelling around was a very ordinary thing as well. However, under the pandemic, there are many restrictions, hence, we have fewer opportunities to meet. I cherish it every time I meet my relatives and friends because we do not know what will happen later. Because of this pandemic, many people actually died, which has made me cherish everything more than before.*

(Student with financial difficulty, group C, F-C-2).

For students’ relationships with the community and society, students stated that the pandemic made them become more concerned about the news or social issues. They understood that they were a part of society, and they tried to show care by staying at home more and helping people in need. Students also showed care to the community by cherishing the opportunity to connect with people in the community. They became more open to communicating with others because it was difficult to have face-to-face communication during the pandemic, as a student explained:

*I’ve come to cherish every opportunity to communicate and connect with others, whether they are strangers, family members, or friends. In the past three years, the pandemic has significantly reduced our chance of face-to-face interaction, so I would cherish these opportunities more in the future.*

(Student who coped well, group B, C-B-7).

### 4.2. Infusing Ordinary Events with Meaning

Another important aspect of meaning-focused coping is infusing ordinary events with meaning. According to Folkman et al. [25], when individuals deliberately infuse their daily lives with positive meaning under stressful situations, it could result in more positive emotions and better well-being during these difficult times. In the current study, it was found that students reported a high level of negative emotions (*n* = 15, 27%) and loneliness (*n* = 8, 14%) during the pandemic. They stated the importance of paying attention to and taking care of their own mental well-being and needs. They would try to relieve the negative emotions by engaging in various activities, as a student stated:

*Another aspect is relieving stress. Due to the heavy academic, personal, and even family pressure during the pandemic, for some stress-relieving activities, for example, finding time to relax, taking walks, sitting outside, looking at the sky, and having moments of quiet reflection, we have more opportunities to deliberately do these things during the pandemic. In the past, we were probably rushing most of the time, like commuting to school, and we didn’t have much time to engage in these stress-relieving activities. This opportunity has arisen during the pandemic.*

(Student who coped well, group A, C-A-7).

Furthermore, during the pandemic, there was a lot of “alone time” for students, so they learned to “have fun alone and think of ways to relieve the negative emotions” (student who coped well, group A, C-A-2). Students engaged in various activities at home. As one student stated, “I have learned the way to face loneliness. During home quarantine, I did many things by myself, for example, cooking, doing housework, and reading” (student who coped well, group C, C-C-6). Students thought that being able to enjoy time alone and take care of themselves demonstrated personal growth. They tried to pay attention to the present moment instead of thinking about the future, which enabled them to fight loneliness during the pandemic, as a student stated:

*I have learned the way to deal with loneliness when I lived alone in the 5th wave of the pandemic. When I had nobody to chat with and all my classmates had their own issues to take care of, I learned to deal with all the problems on my own. To resolve my feeling of loneliness, I only paid attention to the happiness at the moment and did not think about the future. For example, I did not think about my loneliness in the long-term and what to do if there is no company in the future. The experience has taught me how to handle the feeling of loneliness. It is the lesson that the pandemic has taught me.*

(Mainland Chinese student, group B, M-B-6).

### 4.3. Realigning Priorities

Reordering priorities refers to the reappraisal of things that matter to individuals and the reallocation of life priorities when facing stressful situations, which may provide individuals with a new sense of purpose but may also lead to the occurrence of both positive and negative emotions. The pandemic enabled students to cherish the present and plan for the future (*n* = 6, 11%). Students learned to be grateful for what they had and their relationships with family members and friends and not to push themselves too hard, as a student stated:

*The pandemic has given me a lot of time and space to think. I could think clearly about my future directions. For example, in the past, I thought I should meet a lot of people after entering college, or my university life is complete only after achieving the list of ‘Top 5 Things to Do in College’. But now, I think it is good enough for me to attend lectures, complete assignments, enrich myself when I have spare time, and communicate with my friends and have fun together. I think these things are good enough for me already. I realize I should live in the present. I don’t push myself too much to pursue things anymore.*

(Student with financial difficulty, group C, F-C-1).

Witnessing the loss of lives during the pandemic also changed students’ life priorities. They learned the importance of health and taking care of themselves.

Furthermore, students stressed that staying at home enabled them to have more self-reflection (*n* = 13, 23%). Moments of self-reflection gave students opportunities to pause and relax and to think about their past and future development, which were beneficial to their personal growth, as a student stated:

*I think the pandemic has given me a chance to pause and relax. As a student in Hong Kong, I had to compete with others for secondary school places, university places, and good program selection in order to get a good job after graduation, so I was used to rushing. And everyone’s life plan is similar: we tried our best to get into a good secondary school, then a good university, and find a good job. However, I was a bit lost sometimes. I didn’t know what I was doing, or whether the things I was doing is really what I wanted, or I was just going with the flow, that means I was only doing what I was told. The pandemic has really given me a chance to rest. I had some spare time to be alone and think: what am I doing now? What do I want to do in the future? I could also reflect on the bad experience I had. For the bad experience, I could reflect whether the things I did were not good enough. When I face similar situations in the future, how could I do better? So, I think the pandemic has given me a chance to reflect.*

(Student who coped well, group C, C-C-2).

Students also had time to think more and plan for their future education and career, and they changed their life priorities during the pandemic, as a student stated:

*I did reflect on my life, or you could say I reflected my expectation towards the future. I rarely reflect on my past experience because I think there is nothing worth being nostalgic about. I tend to focus on my current life, like how could my major help me in the workplace? Could I find a decent job in the future? Could I get a job [in Hong Kong] after graduation or should I go back to Mainland China? When I consider these questions, I wonder why I have to put pressure on myself. If I can’t live happily now, how can I live happily in the near future? Carpe diem is always right.*

(Mainland Chinese student, group B, M-B-6).

### 4.4. Adaptive Goal Processes

Adaptive goal processes refer to re-evaluating, relinquishing, and substituting unrealistic goals with new and meaningful goals in stressful situations, which may lead to the occurrence of both positive and negative emotions. In the current study, a significant number of participants stressed that the pandemic enabled them to have a more positive attitude towards life (*n* = 9, 16%). By reading news about separation and death during the pandemic, they learned the importance of not taking things for granted and not wasting time, as a student explained:

*The pandemic has made me understand that if I want to do something, I must do it, or I should try it. I should not wait for the time past, or wait for a while before doing it. At the beginning of the pandemic, when there was no vaccine, there were many news about separations and death every day. It made me realize that many things should not be taken for granted and they may be lost within a day. Moreover, during the pandemic, I couldn’t do what I wanted to do, and couldn’t see the people I wanted to see, which have made me realize that time doesn’t wait for anyone. So, I should do the things I want to do because I may not have as much time as I thought. I shouldn’t waste time anymore.*

(Student who coped well, group C, C-C-5).

Moreover, because the pandemic caused many uncertainties in society, students felt that many things seemed impermanent to them. They realized the importance of paying more attention to themselves and gaining control of their own lives. Furthermore, the pandemic also made students realize the importance of independence and personal growth in preparing themselves to face potential challenges in the future, as a student stated:

*The pandemic has made me understand that self-care and independence are very important. It has made me see clearly how cruel and bad this world is. It’s not as good as I previously imagined. So, it is important for me to strive to make myself better, or to enhance [my personal strength], to improve my capacity, and then to handle all kinds of emergencies. Also, I should be financially independent, which is very important. To make more money, so I will have more opportunities and choices in the future. My mindset has experienced growth during the pandemic.*

(Mainland Chinese student, group A, M-A-3).

In addition to working hard, enjoying life was also important to students, and they learned to enjoy life and have fun when they were given opportunities, as a student explained:

*The lesson I have learned from this three-year pandemic is the importance of enjoying life and seizing the present moment. For example, if I want to travel, or if I want to see a concert I like, I should do it now. If I have the opportunity, I should seize it because I never know what will happen next. Before the pandemic, I thought I could have the freedom to travel everywhere, go abroad to have fun, or travel to other cities to watch my favorite performance after my college entrance exams. However, the pandemic started, and it wasn’t until the end of this year that the situation has gotten better slightly, and I could go out to have fun. I feel that I have missed a lot of things in these three years, so I think it is important to seize the opportunity now.*

(Mainland Chinese student, group A, M-A-5).

### 4.5. Benefit Reminding

Benefit reminding is defined as the effortful cognitions individuals make to remind themselves of the possible benefit they could gain from stressful situations, which may lead to the presence of both positive and negative emotions during adversity [19,74]. In the current study, students tried to be optimistic by reminding themselves that the pandemic would end one day (*n* = 2, 3.6%), which enabled them to remain hopeful even during stressful situations, as a student stressed:

*I have learned the way to maintain a positive and optimistic attitude. The pandemic has lasted for a long time, so we have suppressed many negative emotions. And we have observed many negative things happened around us. So, I keep reminding myself that there is still hope for the future.*

(Student who coped well, group C, C-C-6).

### 4.6. Perceived Benefits

According to Tennen and Affleck [19], to regard the discovery of benefits as a coping strategy, it is essential that the discovery leads to individuals finding comfort during adversity. In the current study, students perceived benefits in terms of their personal strengths, interpersonal relationships, and pandemic-related abilities. However, explicit relationships between these perceived benefits and students’ positive emotions were not shown, so these benefits were grouped under a new theme, “perceived benefits”. First, research participants perceived benefits gained in terms of their personal strengths, including enhanced adaptability and flexibility (*n* = 7, 13%), independence (*n* = 7, 13%), problem-solving skills (*n* = 8, 14%), and self-discipline (*n* = 6, 11%). For adaptability and flexibility, because of the frequently changing preventive policies, students needed to change their plans and behaviors constantly during the pandemic, so their adaptability and flexibility were strengthened, as a student described:

*The most significant change [in me] is my adaptability. Because the situation of the pandemic has been changing rapidly, it has led to a frequent change in the preventive measures. It requires individuals to flexibly handle these changes and deal with other external changes. As a result, I need to change my behaviors according to these changes, so my adaptability has been enhanced.*

(Student who coped well, group A, C-A-1).

Moreover, students stated that their problem-solving skills, including the ability to plan and implement, improved during the pandemic because they needed to face different challenges and difficulties. They were forced to think of solutions to various problems, as a student stated:

*For the lesson I have learned, I have learned that people can’t change the larger environment, so they can only improve their abilities, including the ability to make realistic goals, turn long-term goals into short-term goals, and make practical arrangement. Another important ability is the ability to plan. We could not change the pandemic in the larger environment. However, to achieve our own goals, we need to make alternative or backup plans. So, my ability to implement and plan has improved.*

(Mainland Chinese student, group A, M-A-1).

Furthermore, the long period of online learning also strengthened students’ self-discipline and self-learning skills, as a student stated:

*I would say it’s self-discipline because when I am doing an online course, several clicks and YouTube is ready for me. I have to control myself and allocate my time well. That is, if it is the time for me to study, I must study, I can’t let other things distract me. Self-discipline is not only about school work, it is also related to our finance. Controlling my shopping impulse can make my bank finance more stable. So, I have learned that I must be disciplined in all things. I can’t be distracted by other things as I may lose my plan.*

(Student with financial difficulty, group C, F-C-6).

Another aspect of personal strength is independence, which is closely related to problem solving and emotional management skills. Students stressed that they were forced to be alone or to solve problems by themselves during the pandemic, which enabled them to be more independent, as a student stated:

*Because I had to live in a relatively confined place by myself [during the pandemic] and it was also a relatively unfamiliar environment for me, I must improve my independence, that is, to learn to solve problems by myself, and not relying on my family anymore. I would only tell them about my problems after solving them. I have already acquired this ability, that means I am starting to be mature.*

(Mainland Chinese student, group A, M-A-6).

Second, the pandemic strengthened students’ skills that were specific to the pandemic, including learning to seek help and use online platforms (*n* = 5, 9.0%), gaining more understanding of the pandemic, and gaining increased hygiene awareness (*n* = 3, 5.4%), as a student stated:

*Because a lot of things have switched to online mode, I have learned more about the way to use online tools, for example, online meeting platforms. I could apply these skills later, including in group projects and in my future jobs. There are other online resources. I have learned the way to use my home department’s Virtual Lab. In the future, no matter it is a pandemic or not, if I can’t do face-to-face laboratory practice, I can still use Virtual Lab to practice laboratory skills at home.*

(Student who coped well, group A, C-A-4).

Third, when facing challenges during the pandemic, some students had more opportunities to communicate with their family members and friends, which improved their relationships (*n* = 2, 3.6%), as a student stated:

*The pandemic has made me gain better understanding of the way to get along with my family members. Now, I have more understanding of the way I used to think, and have more ideas of effective communication. In the past, I didn’t have much communication with my family members. However, because of the pandemic, we were a bit scared, so we started to communicate more and have more understanding of each other and the way we think. When we fell into a family financial crisis, we discussed the reasons and the way to help each other.*

(Student with financial difficulty, group C, F-C-7).

## 5. Discussion

The current study employed the Revised Stress and Coping Model as the theoretical framework to explore Hong Kong university students’ meaning-focused coping amid the COVID-19 pandemic. The study found that university students used the five meaning-focused coping strategies, including “benefit finding”, “infusing ordinary events with meaning”, “realigning priorities”, “adaptive goal processes”, and “benefit reminding” [25,26]. The current study also identified a new theme, “perceived benefits”, which refers to the benefits that did not lead to a comforting effect during difficult times and were regarded as “benefit finding as a conclusion” rather than a coping strategy [75]. In the literature, some studies have applied the Transactional Model of Stress and Coping as the theoretical framework to explore individuals’ meaning-focused coping during the COVID-19 pandemic. For example, a phenomenological study on US perinatal women showed that meaning making was achieved through connection finding, emphasizing gratitude, and recognizing opportunities for change [76]. A study on US college students also identified various self-related and societal benefits during the pandemic [12]. For post-traumatic growth, a study on Mainland Chinese college students revealed that their post-traumatic growth comprised four core dimensions, including reflections related to society and the country, changes in self-awareness, more social behavior, and changes in lifestyle [10]. As there are few studies examining the five categories of meaning-focused coping during the COVID-19 pandemic, this study is innovative in a Chinese context.

**Figure 2:**
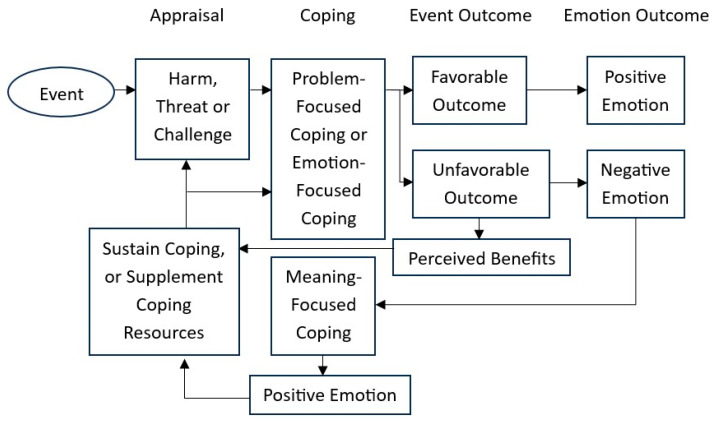
Conceptual framework of the current study.

Based on the research findings of the current study, a conceptual framework was developed (Figure 2). A new theme, “perceived benefits”, was added, which denoted the benefits that did not lead to the generation of positive emotions during adversity. The current study further suggests that these perceived benefits could help individuals to sustain coping and restore resources. This argument is further supported by empirical studies in the literature. Lancastle and Boivin’s [77] investigation of women waiting for an in vitro fertilization (IVF) pregnancy test confirmed the effectiveness of a positive reappraisal coping intervention over a positive self-affirmative (positive mood) intervention. The positive reappraisal coping intervention helped participants sustain coping efforts. Furthermore, studies on HIV/AIDS patients have also shown that positive reappraisal could enhance individuals’ efforts in health promotion and self-management of the disease [78]. Also, positive psychological beliefs, including meaning, control, and optimism, were regarded as resources for individuals to maintain their mental and physical well-being amid the pandemic [79]. Future studies could further investigate the role of positive reappraisal and positive psychological beliefs in sustaining coping and restoring individuals’ resources during difficult times.

### 5.1. Finding Benefits in Adversity

Out of the 56 participants, the current study found a significant number of participants perceiving benefits from the pandemic, which led to the generation of positive affect during adversity (*n* = 35, 63%). Positive affect includes being more accepting, flexible, and open-minded and worrying less (*n* = 11, 20%), having an increased sense of responsibility and self-perceived personal growth (*n* = 4, 7.1%), cherishing relationships with family members and friends (*n* = 19, 34%), and being more open to communicating with strangers and showing care to society (*n* = 8, 14%). Findings from the current study show consistency with other studies conducted during the pandemic. A qualitative study conducted by Sun et al. [80] found “cherishing life and family” in COVID-19 patients in Mainland China, and it was regarded as psychological growth and outlook. Also, open-mindedness has been regarded as a character strength, and it was found to be positively related to Indonesian college students’ well-being during the pandemic [81]. Studies conducted in both Eastern and Western contexts have shown adolescents’ or young adults’ increased care-taking responsibilities for family members during the pandemic. For example, a qualitative study on US university students revealed that students from working-class families had more responsibilities for their own and their family members’ well-being during the pandemic [82]. Studies conducted in Eastern contexts showed similar findings. A qualitative study on adolescents in Italy, Lebanon, Singapore, and the United Kingdom showed that young people were more aware of their role in reducing parents’ pressure and decreasing the negative impact of the pandemic on their families, including caring for sick parents and caring for and providing home-schooling for younger siblings [83]. A few independent studies have also stressed the increased household chores among Bangladeshi rural students [84] and Indian children [85] amid the pandemic.

However, some studies have revealed that family or caregiving responsibilities were associated with poor mental health in US undergraduate students [86], Italian young adult carers [87], and Bangladeshi students [88] during the pandemic. But, other studies have shown that household chores were associated with reduced mental distress in Bangladeshi university students [89] and Chinese college students [90] during home confinement. Also, the literature shows mixed results regarding young people’s prosocial behavior and their well-being. More COVID-19 prosocial behaviors were related to increased levels of anxiety and burdensomeness in US adolescents [91], but helping others was associated with increased happiness in Dutch adolescents [92]. Empathic concern and prosocial attributes also decreased significantly in both Mainland Chinese adolescents [93] and Dutch adolescents [94] during the pandemic because of the decreased opportunities for prosocial actions. Hence, more studies on the impact of such “benefits” on the psychological well-being of individuals during the pandemic should be further explored.

In addition, when comparing the three categories of students in the current study, a higher percentage of students facing financial difficulties had an increased sense of responsibility and self-perceived personal growth (four out of four participants, 100%), and being more open to communicating with strangers and showing care to society (4 out of 8 participants, 50%). These findings echo another study conducted on students from an elite US university, showing that students from upper-middle-class families could obtain rich material support and guidance from their parents, while students from working-class families needed to take more responsibility for their own well-being or to take care of their family members [82]. However, for prosocial behavior, the findings of the current study are not consistent with the findings from the literature. A study showed that sociodemographic factors did not predict prosocial behavior in 78 countries during the pandemic [95], while another study showed that pro-sociality was low for French adolescents with low socioeconomic status before the pandemic and that COVID-19 infections within families further decreased adolescents’ pro-sociality [96]. The current study supports the finding that students facing financial difficulty experienced increased family responsibilities during the pandemic, which enhanced their personal growth. These students also exhibited more prosocial behavior, which led to the generation of positive emotions in them during the pandemic.

### 5.2. Infusing Daily Lives with Positive Meaning

A significant number of participants in the current study deliberately infused their daily lives with positive meaning, which resulted in more positive emotions. They tried to deal with their negative emotions by accepting the self (*n* = 15, 27%) and learning to deal with loneliness (*n* = 8, 14%). In the literature, researchers have distinguished the difference between the generation of global meaning and situational meaning when coping with stressful experiences. Global meaning refers to a person’s basic goals, assumptions, and beliefs about the world, while situational meaning refers to the interaction between a person’s global beliefs and his/her circumstances [27]. Examining earthquake survivors, Gan et al. [97] revealed that changes in situational meaning were more important than changes in global meaning in predicting their psychological adjustment. In terms of stress and coping, situational meaning refers to a person appraising his/her interaction with the environment, which influences the coping process and outcome [24]. The essence of meaning management for a person is to reduce the difference between his or her global meaning and the appraised meaning of a situation. The current study contributes to the literature by demonstrating how young people appraised the pandemic as a chance for developing their abilities to fight against negative emotions and loneliness, which led to the reduction of these negative emotions.

In addition, empirical studies conducted during the pandemic have revealed the significant relationship between infusing daily lives with positive meaning and emotional well-being. In the UK, Ooi et al. [98] showed that young people engaged in self-fulfilling activities and utilized strategies to regulate their thoughts and emotions, which led to self-perceived personal growth. For Italian adolescents, Fioretti et al. [99] found that adolescents found pleasure spending time by themselves and doing activities that were interesting to them, e.g., reading, listening to music, and painting, which led to greater acceptance of the self. Among US adults, Kim et al. [100] revealed a positive association between well-being and life meaning, coping style, and self-transcendent wisdom, showing the importance of self-transcendence to a deeper sense of well-being. The current study contributes to the literature by illustrating how young people in Hong Kong infused their daily activities with positive meaning, which led to better emotional well-being.

Furthermore, when comparing the three categories of students in the current study, a higher percentage of “students who coped well” accepted the self and dealt with negative emotions (8 out of 15 participants, 53%), which led to the generation of positive emotions during difficult times. In the literature, studies have identified significant relationships between self-acceptance, emotion regulation, and resilience in young people during and after the pandemic. Examining Italian adolescents, Perasso et al. [101] found that adolescents’ self-efficacy in regulating emotions was a significant predictor of their resilience in the post-pandemic period. Furthermore, Yao et al. [102] found that self-acceptance and psychological capital positively contributed to Chinese university students’ well-being during the pandemic. Additionally, Brites et al. [103] showed that university students with high levels of self-efficacy were more likely to successfully cope with challenges and had less mental distress during the pandemic. Findings from the current study echo the existing literature and qualitatively demonstrate the significant relationship between resilience, self-acceptance, and emotion regulation in Hong Kong university students during the pandemic.

### 5.3. Reordering Life Priorities

A significant number of participants in the current study reordered their life priorities during the pandemic, which involved the reappraisal of things that were important to them. The reordering of life priorities was related to better emotional well-being. The participants cherished the present and had new plans for the future (*n* = 6, 11%). They engaged in more self-reflection, which led to personal growth and future planning (*n* = 13, 23%). Scholars have argued that in addition to situational meaning, global meaning is also important in the stress and coping process [27]. One of the dimensions of global meaning is related to a person’s beliefs about the world, the self, and the self in the world. Changes in global meaning may affect a person’s life priorities. The current study contributes to the literature by illustrating how the pandemic experience enabled university students to cherish and utilize time during the pandemic for self-reflection, which led to personal growth and better future planning.

Moreover, empirical studies conducted during the pandemic have also revealed the importance of changes in life priorities for individuals’ personal growth. The global scoping review conducted by Carey et al. [104] showed that adolescents had increased social awareness and feelings of uncertainty, and they changed their short- and long-term plans for educational and career development during the pandemic. For Canadian and American adults, Asmundson et al. [105] showed that changing life priorities and having a greater sense of self-reliance were important parts of an individual’s personal growth during the pandemic. However, some studies found that the value priorities of young people remained relatively stable during the pandemic because of the assumed temporality of the pandemic [106,107]. The current study contributes to the literature by qualitatively presenting the changed priorities and self-perceived personal growth of Hong Kong university students during the pandemic.

In addition, when comparing the three categories of students in the current study, a significantly higher percentage of “students who coped well” had increased frequency of self-reflection, which led to personal growth and future planning (10 out of 13 participants, 77%). In the literature, studies have highlighted the significant association between resilience and self-reflection. A theoretical model suggested that self-reflection on daily stressors could strengthen individuals’ resilient capacities [108]. Coping insights resulting from self-reflection could also enhance individuals’ resilient capacities [109]. Furthermore, Taylor et al. [79] argued that self-care practices and self-compassion were crucial to undergraduate nursing students’ resilience and well-being during the pandemic. The current study’s findings align with findings from the literature, highlighting the significant relationship between self-reflection, resilience, and mental health among young people during the pandemic.

### 5.4. Changing Life Goals to Adapt to Challenges

Participants in the current study had undergone adaptive goal processes by substituting unrealistic goals with new and meaningful goals, which led to improved well-being. They had more positive attitudes towards life, including being more proactive and independent, gaining more self-control, and enjoying life (*n* = 9, 16%). Built on the Transactional Model of Stress and Coping, Park and Folkman [27] elaborated the dimensions of global meaning in the stress and coping process. One of the dimensions of global meaning is the motivational dimension, which affects a person’s life goals and purpose. Meaning could motivate or direct the striving of a person. This kind of meaning is typically represented as a goal. Distal goals offer people a sense of purpose, while proximal goals may include daily activities that vary between people. The current study contributes to the literature by showing that Hong Kong university students developed more positive attitudes towards life in various dimensions, which is related to their distal goals and sense of purpose.

Studies conducted during the pandemic have also revealed findings similar to those of the current study. A systematic review of higher education institution students’ growth mindset found a positive contribution to the academic performance and mental health of underrepresented students [110]. A growth mindset comprises various skills that are interdependent, including self-control, self-efficacy, and self-esteem. Qualitative studies on undergraduate students have also revealed that the desire to succeed and motivation were the intrinsic factors that enhanced students’ resilience during the pandemic [111], and strengthened resilience could help students adapt to the changing world [112]. The current study contributes to the literature by qualitatively demonstrating how Hong Kong university students underwent adaptive goal processes by enhancing their growth mindset and independence during the pandemic.

In addition, when comparing the three categories of students in the current study, a higher percentage of Mainland Chinese students applied adaptive goal processes (five out of nine participants, 56%), including being more proactive and independent, gaining more self-control, and enjoying life. This finding could be explained by the special life goals and mentality of Mainland Chinese students studying in Hong Kong. A study on Mainland Chinese students studying in Hong Kong found that autonomous motivation significantly predicted their adaptation outcomes [113]. Another study on Mainland Chinese students studying in Hong Kong also highlighted the important protective role of sense-making coping, which is a form of meaning-focused coping, in students’ psychological adjustment when studying in Hong Kong [114]. For Hong Kong university students, there are limited studies on their life goals and mentality. One of the exceptions is the study conducted by Ge et al. [115], which shows the positive association between Hong Kong university students’ internal locus of hope (hope regarding one’s own capabilities) and their academic outcomes. Findings from the current study add to the existing literature, stressing the importance of independence in Mainland Chinese students’ mentality, which was related to their meaning-focused coping when studying in Hong Kong during the pandemic.

### 5.5. Reminding Benefits Gained During Adversity

Participants in the current study used “benefit reminding” to cope with stress during the pandemic, which is the cognitive effort individuals make to remind themselves of the possible benefit they could gain from stressful situations. Students tried to remind themselves that the pandemic would end one day (*n* = 2, 3.6%) so that they could remain hopeful, even when facing adversity. Folkman [26] highlighted the blurry distinction between benefit reminding and benefit finding, which is influenced by the timepoint during which the variable is measured during the stress process. The current study contributes to the literature by showing that university students reminded themselves of the temporary nature of the pandemic, which strengthened their hope during difficult times. Other empirical studies conducted during the pandemic have also shown similar findings. Examining young people in Peru, Javier-Aliaga et al. [116] found that hope and resilience were protective factors in relation to COVID-19 fear. Studying Hong Kong university students, Sun et al. [117] revealed that hope and mindfulness were negatively and longitudinally related to their internalizing and externalizing behaviors.

### 5.6. Perceiving Benefits Gained During Adversity

The current study found that a significant number of students perceived benefits related to their improved interpersonal relationships, enhanced personal strengths, and heightened skills specific to the pandemic (*n* = 30, 54%). Because these benefits did not explicitly lead to the generation of positive emotions in the participants, they were not classified as coping strategies, and related quotations were grouped under a new theme called “perceived benefits”. The current study contributes to the literature by qualitatively illustrating the perceived benefits Hong Kong university students gained from the pandemic. In the literature, studies have also revealed that individuals gained benefits in various areas during the pandemic. Gander and Wagner [118] found that German adults perceived positive changes in most of their character strengths, with the biggest improvements in gratitude, appreciation of beauty and excellence, humility, and prudence. Based on an analysis of the resilience literature, Shek [119] highlighted that COVID-19 stress and experience may positively contribute to adolescents’ problem-solving skills. A qualitative study on university students also found that students used self-discipline strategies to overcome remote learning challenges during the pandemic [120]. The systematic review conducted by Yu [121] showed students’ improvement in digital literacy and its importance in improving their online learning achievements.

However, findings from the current study differ from some research results in the literature. The systematic review conducted by Stavridou et al. [122] showed that young people stayed at home more often and lost daily communication with peers during the pandemic. An empirical study on US college students also revealed that young people faced difficulties in maintaining affective connections through online platforms such that physical distancing during the pandemic created emotional distancing with their peers [123]. The contradiction could be explained by the fact that only a small number of participants perceived improved relationships with family members and friends in the current study.

In addition, when comparing the three categories of students in the current study, significantly more “students who coped well” had enhanced adaptability and flexibility (six out of seven participants, 86%) and strengthened problem-solving skills during the pandemic (five out of eight participants, 63%). In the literature, studies have highlighted the significant relationships amongst resilience, adaptability, flexibility, and problem solving in young people during the pandemic. Brammer [124] showed that US health science students’ growth mindset (i.e., viewing challenges as learning opportunities) during the pandemic was commonly seen in students who were more resilient. Also, Herbers et al. [125] emphasized the importance of adaptive systems in children during the pandemic. It was argued that children showed resilience to disaster when their adaptive systems were performing effectively, which depended on their developmental histories and resources provided by the community or society. Furthermore, studying Turkish high school students, İme and Ümmet [126] revealed the significant association between their emotional flexibility, subjective well-being, and resilience during the pandemic. Examining South African university students, Pretorius and Padmanabhanunni [127] argued that students’ positive perception of their problem-solving skills could be a resistance resource for their well-being during the pandemic. Findings from the current study add to the literature, demonstrating that resilient young people perceived more benefits in terms of their adaptability, flexibility, and problem-solving skills during the pandemic.

Furthermore, the current study found that significantly more Mainland Chinese students had enhanced independence (five out of seven participants, 71%). This finding echoes the findings of another study on Mainland Chinese students studying in Hong Kong, highlighting the important role of their autonomous motivation in positive adaptation when studying in Hong Kong [113].

### 5.7. Theoretical Implications

The findings of this study have several theoretical implications. First, the current study supports and extends the Revised Stress and Coping Model. The integrated review of the meaning literature conducted by Park [13] found that theories on meaning and meaning making were well-developed, but there was limited empirical research to test these theories. The current study fills this knowledge gap by empirically supporting the five categories of meaning-focused coping proposed by Folkman [26]. Second, this qualitative study contributes to the COVID-19 literature by capturing the application of meaning-focused coping and perceived benefits of young people in Hong Kong using their own words. Third, data for the current study were collected from late December 2022 to mid-January 2023, when Hong Kong was beginning to return to normal by loosening some of the pandemic restrictions, including nucleic acid testing and social distancing measures for people entering Hong Kong, while also maintaining some preventive measures, such as the mask order, the quarantine order for COVID-19 patients, and travel restrictions between Hong Kong and Mainland China. So, the current study enables us to understand young people’s retrospective view of their use of meaning-focused coping during the pandemic and their perceived benefits towards the end of prolonged pandemic restrictions in Hong Kong. Fourth, the current study fills the knowledge gap by examining the coping of university students in three categories, namely, students who coped well, students with financial difficulties, and Mainland Chinese students studying in Hong Kong, which have not been thoroughly studied in the COVID-19 literature.

### 5.8. Practical Implications

For practical implications, the current study provides evidence of the use of meaning-focused coping and benefits gained by young people during the pandemic, which allows us to understand the utilization of related coping strategies and the positive impact of this global disaster on young people. This could facilitate better supporting of young people’s healthy development in the post-pandemic period. Moreover, most interventions related to stress coping have not paid enough attention to positive meaning and emotions. Given the reciprocal relationship between positive emotions and positive coping [128], future interventions and policies related to stress coping could focus on the regulation of emotions to enhance their effectiveness. Furthermore, the current study could contribute to fostering meaning making and sense making across the general population after the pandemic, which may benefit psychological balance and well-being [14]. The current study highlights the importance of spirituality to young people’s well-being. Existing positive youth development programs could play a crucial role in helping young people enhance their spirituality [129,130].

### 5.9. Limitations

Although the present study generates innovative research findings, it also has limitations. First, because of the small sample size of the qualitative study, the findings may only be applicable to the research participants in the current study. However, according to the naturalistic generalizability principle in qualitative study [131,132], readers may determine whether the findings could be generalized to university students in other contexts based on readers’ related experiences and the thick description of the phenomenon in the current study. Second, the study only interviewed research participants at one timepoint during the three-year pandemic restriction in Hong Kong, so young people’s longitudinal changes in their use of meaning-focused coping and perceived benefits were not sufficiently examined. A longitudinal study design could be adopted in future research to examine related changes over a certain period of time. Third, the current study applied the deductive analytical approach (i.e., top–down) based on the five categories of meaning-focused coping. Although a new theme (i.e., perceived benefits) was identified, the analytical process was mainly based on this theoretical framework, while the interactions between themes and the discovery of new themes were not the main emphases. Future studies could apply an inductive approach or a mix of inductive and deductive approaches in qualitative research to explore new themes in meaning-focused coping. Finally, the influence of social factors on university students’ meaning-focused coping was not sufficiently examined in the current study. Social factors may affect young people’s prosocial behavior and the generation of positive emotions during adversity [133]. Future studies on meaning-focused coping could further explore various social factors.

## 6. Conclusions

The current study applied a deductive approach to thematic analysis to examine the use of meaning-focused coping and perceived benefits among Hong Kong university students during the pandemic. The results showed that students employed the five forms of meaning-focused coping in the Revised Stress and Coping Model, including “benefit finding”, “infusing ordinary events with meaning”, “realigning priorities”, “adaptive goal processes”, and “benefit reminding”. Additionally, students found benefits that were not explicitly related to the generation of positive emotions in difficult times, including enhanced interpersonal relationships, increased personal strengths, and reinforced skills related to the pandemic. This paper revealed how positive meaning could help young people cope with adversity by increasing their positive emotions. The study also found that the pandemic has brought various positive benefits to young people in Hong Kong. Furthermore, the differences in the use of meaning-focused coping and perceived benefits by three groups of university students (i.e., students with family or personal financial difficulties, students who coped well, and Mainland Chinese students studying in Hong Kong) were compared and discussed.

## Figures and Tables

**Table 1:** Three categories of research participants, related questions asked before the focus group interviews, participant codes, and the number of participants in each category.

Categories	Related Questions Asked Before the Focus Group Interviews	Participant Codes and Number of Participants
Students with financial difficulties	Did you or your family face financial difficulties during the pandemic?	F-A-2, F-A-3, F-A-4, F-A-6, F-B-1, F-B-2, F-B-4, F-B-6, F-B-7, F-B-8, F-C-1, F-C-2, F-C-3, F-C-4, F-C-5, F-C-6, F-C-7, F-C-8, F-C-9 (*n* = 19)
Students who coped well	Despite the difficulties I have faced during the pandemic, I consider myself to have coped well. Do you agree with this statement?	C-A-1, C-A-2, C-A-4, C-A-5, C-A-6, C-A-7, C-B-1, C-B-2, C-B-3, C-B-4, C-B-5, C-B-6, C-B-7, C-C-1, C-C-2, C-C-3, C-C-4, C-C-5, C-C-6, C-D-1, C-D-2, C-D-4 (*n* = 22)
Mainland Chinese students studying in Hong Kong	Are you a local, mainland, or international student?	M-A-1, M-A-2, M-A-3, M-A-5, M-A-6, M-A-7, M-A-8, M-B-4, M-B-5, M-B-6, M-B-8, M-B-9, M-C-4, M-C-5, M-C-7 (*n* = 15)
Total number of participants		56

**Table 2:** Summary of the themes and sub-themes.

Meaning-Focused Coping and Perceived Benefits	Sub-Themes
Realigning priorities (*n* = 19)	Cherished the present and planned for the future (*n* = 6)
	Had more self-reflection, leading to personal growth and future planning (*n* = 13)
Adaptive goal processes (*n* = 9)	Had a more positive attitude towards life, including being more proactive and independent, gaining more self-control, and enjoying life (*n* = 9)
Benefit finding (*n* = 35)	Were more accepting, flexible, and open-minded and worried less (*n* = 11)
	Increased sense of responsibility and self-perceived personal growth (*n* = 4)
	Cherished relationships with family members and friends (*n* = 19)
	Were more open to communicating with strangers and showing care to society (*n* = 8)
Benefit reminding (*n* = 2)	Maintained hope (*n* = 2)
Infusing ordinary events with meaning (*n* = 23)	Accepted self and dealt with negative emotions (*n* = 15)
	Learned to deal with loneliness (*n* = 8)
Perceived benefits (*n* = 30)	Enhanced adaptability and flexibility (*n* = 7)
	Enhanced independence (*n* = 7)
	Strengthened problem-solving skills (*n* = 8)
	Strengthened self-discipline (*n* = 6)
	Learned to seek help and use online platforms (*n* = 5)
	Increased hygiene awareness (*n* = 3)
	Improved relationship with family members and friends (*n* = 2)

## Data Availability

The data supporting the findings of this study are available upon request from the corresponding author. The data are not publicly available due to ethical concerns regarding the publication of focus group interview transcripts in a public repository. Specifically, when the data were collected, participants only gave consent for the findings to be published anonymously for educational and research purposes. Making the data public would violate this consent.

## Supplementary Materials

The following supporting information can be downloaded at https://www.mdpi.com/article/10.3390/ijerph22040614/s1, Table S1: Additional illustrative quotes.

## Abbreviations

The following abbreviations are used in this manuscript:

COVID-19	Coronavirus disease 2019
PANAS	Positive and Negative Affect Schedule

## Appendix A

### Appendix A.1. Reflexivity Statement

The first author is a researcher who studies mental health and leadership education of young people, focusing generally on qualitative research. She has conducted more than 50 individual and focus group interviews with young people and secondary school teachers on mental health, teaching and learning effectiveness, and leadership education. She is well-equipped with qualitative research skills and can design qualitative studies and analyze related data rigorously. Yet, the first author’s potential bias is that she was not an insider of the group being investigated (i.e., university students), so research participants may not be fully open when communicating their feelings and experience in the interviews. This bias was minimized by building rapport with the research participants before and during the interviews.

The second author is a psychologist who has contributed to research on youth development for many years. He has rich experience in conducting both qualitative and quantitative research on various topics, including positive youth development, family process, scale development, quality of life, program evaluation, addiction, and spirituality. His related expertise and experience significantly improved the research design, theoretical framework conceptualization, data analysis, and research findings’ interpretation in the current study. During data analysis, the second author served as a “critical friend” to the first author by challenging the codes’ and themes’ development, which has strengthened the validity and reliability of the current research.

### Appendix A.2. Qualitative Interview Questions

A. Challenges faced by students
1. Can you tell me about the challenges you encountered under the COVID-19 pandemic? Please share your experience.
a. Academic Domain
i. Unfulfilled personal & professional goals due to suspension of learning activities
ii. Online learning
iii. Disruption of outbound activities
iv. Uncertainty on career and employment
v. University life & sense of belonging
vi. Technology literacy related to online learning
vii. Uncertainty on academic development
b. Personal (including physical, psychological, social and spiritual) Domain
i. Physical health (e.g., illness, lack of exercise, dry eyes, sleep problems, body pain)
ii. Psychological (e.g., negative emotions, feeling nervous/anxious/worries/depress/hopelessness, fear of going out, bored, fatigue)
iii. Social (e.g., loneliness, lack of peer support, friendship, peer relationship, organization)
iv. Spiritual (e.g., reflection on/rethinking life, human relationship, living, etc.)
v. Have you reflected on your life meaning during the pandemic?
c. Family Domain
i. Competition to use family resources (e.g., Wifi, space, furniture)
ii. Increased family conflict
iii. Financial hardship (e.g., unemployed/underemployed/business close down/rent/loan)
iv. Role change (e.g., take care of siblings/elder/sick family member when learning)
v. Family members/relatives/friends suffering from the COVID-19
d. Community Domain
i. Discrimination (e.g., test-positive/under quarantine/travel history)
ii. Community sentiment (e.g., measures and policies imposed by the government, performance of officials/members of Legco, experts)
e. Other examples of challenged experienced by the interviewees
B. Coping with Challenges
1. How did you deal with the challenge(s) and the feeling(s) (e.g., stress, negative feelings)? Why did you choose this way? Was it useful? Please share with us your experience.
C. Review
1. Do you think the pandemic has helped you to grow better? Why or why not?
2. What lessons have you learned from the pandemic?

**Table A1:** Summary of the use of meaning-focused coping strategies and perceived benefits among research participants.

Meaning-Focused Coping and Perceived Benefits	Sub-Themes	Participant Codes
Realigning priorities (*n* = 19)	Cherished the present and planned for the future (*n* = 6)	C-B-7, F-A-3, F-C-1, M-A-2, M-C-4, M-C-5
	Had more self-reflection, leading to personal growth and future planning (*n* = 13)	C-A-1, C-A-2, C-A-4, C-A-5, C-A-6, C-B-1, C-C-2, C-D-1, C-D-2, C-D-4, F-A-2, F-A-4, M-B-6
Adaptive goal processes (*n* = 9)	Had a more positive attitude towards life, including being more proactive and independent, gaining more self-control, and enjoying life (*n* = 9)	C-B-4, C-C-5, F-A-2, F-B-7, M-A-2, M-A-3, M-A-5, M-A-7, M-C-7
Benefit finding (*n* = 35)	Were more accepting, flexible, and open-minded and worried less (*n* = 11)	C-B-3, C-B-6, C-C-2, C-C-3, C-C-4, F-A-6, F-B-8, F-C-3, F-C-5, F-C-9, M-B-5
	Increased sense of responsibility and self-perceived personal growth (*n* = 4)	F-A-2, F-A-6, F-B-2, F-C-3
	Cherished relationships with family members and friends (*n* = 19)	C-B-1, C-B-2, C-B-3, C-C-5, C-C-6, C-D-1, C-D-2, C-D-4, F-A-4, F-B-6, F-C-2, F-C-4, F-C-5, M-A-2, M-A-3, M-B-4, M-B-6, M-B-9, M-C-4
	Were more open to communicating with strangers and showing care to society (*n* = 8)	C-B-6, C-B-7, F-A-2, F-A-3, F-B-1, F-B-7, M-B-9, M-C-7
Benefit reminding (*n* = 2)	Maintained hope (*n* = 2)	C-B-6, C-C-6
Infusing ordinary events with meaning (*n* = 23)	Accepted the self and dealt with negative emotions (*n* = 15)	C-A-7, C-B-1, C-B-4, C-B-5, C-B-6, C-B-7, C-D-2, C-D-4, F-A-2, F-A-4, F-B-4, F-C-5, F-C-8, M-A-7, M-B-8
	Learned to deal with loneliness (*n* = 8)	C-A-2, C-A-4, C-C-2, C-C-3, C-C-6, M-A-2, M-B-6, M-C-5
Perceived benefits (*n* = 30)	Enhanced adaptability and flexibility (*n* = 7)	C-A-1, C-A-6, C-B-1, C-B-7, C-C-3, C-C-6, M-B-9
	Enhanced independence (*n* = 7)	C-C-1, C-C-6, M-A-1, M-A-6, M-B-8, M-B-9, M-C-5
	Strengthened problem-solving skills (*n* = 8)	C-A-5, C-A-6, C-B-2, C-C-5, C-D-2, F-A-6, M-A-1, M-B-6
	Strengthened self-discipline (*n* = 6)	C-A-7, C-C-2, C-C-4, F-A-2, F-C-6, M-B-4
	Learned to seek help and use online platforms (*n* = 5)	C-A-2, C-A-4, C-B-5, C-B-6, C-B-7
	Increased hygiene awareness (*n* = 3)	C-C-1, C-C-3, C-C-5
	Improved relationships with family members and friends (*n* = 2)	C-A-6, F-C-7

## References

[1] Guo M., Gan Y., Tong J. (2013). The role of meaning-focused coping in significant loss. Anxiety Stress Coping.

[2] Ward S., Womick J., Titova L., King L. (2023). Meaning in life and coping with everyday stressors. Personal. Soc. Psychol. Bull..

[3] Cohrdes C., Pryss R., Baumeister H., Eicher S., Knoll N., Hölling H. (2023). Support- and meaning-focused coping as key factors for maintaining adult quality of life during the COVID-19 pandemic in Germany. Front. Public Health.

[4] Lau B.H.-P., Chan C.L.-W., Ng S.-M. (2020). Self-compassion buffers the adverse mental health impacts of COVID-19-related threats: Results from a cross-sectional survey at the first peak of Hong Kong’s outbreak. Front. Psychiatry.

[5] Waters L., Allen K.-A., Arslan G. (2021). Stress-related growth in adolescents returning to school after COVID-19 school closure. Front. Psychol..

[6] Eisenbeck N., Carreno D.F., Wong P.T.P., Hicks J.A., María R.-R.G., Puga J.L., Greville J., Testoni I., Biancalani G., López A.C.C. (2022). An international study on psychological coping during COVID-19: Towards a meaning-centered coping style. Int. J. Clin. Health Psychol..

[7] Park C.L., Finkelstein-Fox L., Russell B.S., Fendrich M., Hutchison M., Becker J. (2021). Psychological resilience early in the COVID-19 pandemic: Stressors, resources, and coping strategies in a national sample of Americans. Am. Psychol..

[8] Chu G.M., Goger P., Malaktaris A., Lang A.J. (2022). The role of threat appraisal and coping style in psychological response to the COVID-19 pandemic among university students. J. Affect. Disord. Rep..

[9] Li Q., Hu J., Wan P. (2023). Serial multiple mediation of the relationship between positive coping style and post-traumatic growth among Chinese college students in the aftermath of COVID-19. Int. J. Ment. Health Promot..

[10] Ma Y., Wang H., Chai H., Zhu J., Lin X., Huang H., Sun Z. (2024). Chinese college students’ post-traumatic growth during the COVID-19: A grounded theory study. Humanit. Soc. Sci. Commun..

[11] Xie J.-Q., Zhang H., Zhang X., Yin M.-Z., Yang J., Chen K., Xiong J.-R., Chen Y.-Q. (2022). The mediating role of personal values between COVID-19-related posttraumatic growth and life satisfaction among Chinese college students: A two-wave longitudinal study. Front. Psychol..

[12] August R., Dapkewicz A. (2020). Benefit finding in the COVID-19 pandemic: College students’ positive coping strategies. J. Posit. Sch. Psychol..

[13] Park C.L. (2010). Making sense of the meaning literature: An integrative review of meaning making and its effects on adjustment to stressful life events. Psychol. Bull..

[14] Castiglioni M., Gaj N. (2020). Fostering the reconstruction of meaning among the general population during the COVID-19 pandemic. Front. Psychol..

[15] Linley P.A., Joseph S. (2004). Positive change following trauma and adversity: A review. J. Trauma. Stress.

[16] Park C.L., Fenster J.R. (2004). Stress-related growth: Predictors of occurrence and correlates with psychological adjustment. J. Soc. Clin. Psychol..

[17] Tedeschi R.G., Calhoun L.G. (1996). The posttraumatic growth inventory: Measuring the positive legacy of trauma. J. Trauma. Stress.

[18] Carver C.S. (1998). Resilience and thriving: Issues, models, and linkages. J. Soc. Issues.

[19] Tennen H., Affleck G., Snyder C.R., Lopez S.J. (2002). Benefit-finding and benefit-reminding. Handbook of Positive Psychology.

[20] Frankl V.E. (2006). Man’s Search for Meaning.

[21] Asberg K.K., Bowers C., Renk K., McKinney C. (2008). A structural equation modeling approach to the study of stress and psychological adjustment in emerging adults. Child Psychiatry Hum. Dev..

[22] Ano G.G., Vasconcelles E.B. (2005). Religious coping and psychological adjustment to stress: A meta-analysis. J. Clin. Psychol..

[23] Lazarus R.S., Kanner A.D., Folkman S., Plutchik R., Kellerman H. (1980). Emotions: A cognitive-phenomenological analysis. Theories of Emotion. Vol 1. Emotion: Theory, Research, and Experience.

[24] Lazarus R.S., Folkman S. (1984). Stress, Appraisal, and Coping.

[25] Folkman S. (1997). Positive psychological states and coping with severe stress. Soc. Sci. Med..

[26] Folkman S. (2008). The case for positive emotions in the stress process. Anxiety Stress Coping.

[27] Park C.L., Folkman S. (1997). Meaning in the context of stress and coping. Rev. Gen. Psychol..

[28] Fu Y., Law Y.W. (2018). Chinese adolescents’ meaning-focused coping with prolonged parent-child separation. J. Adolesc. Res..

[29] Watanabe H. (2019). Association between benefit-finding and identity development in adolescence: Are there benefit-finding domains related to high identity achievement?. Int. J. Adolesc. Youth.

[30] Gruszczyńska E., Knoll N. (2015). Meaning-focused coping, pain, and affect: A diary study of hospitalized women with rheumatoid arthritis. Qual. Life Res..

[31] Gumuchian S.T., Peláez S., Delisle V.C., Carrier M.-E., Jewett L.R., El-Baalbaki G., Fortune C., Hudson M., Körner A., Kwakkenbos L. (2018). Understanding coping strategies among people living with scleroderma: A focus group study. Disabil. Rehabil..

[32] Krok D., Telka E. (2019). The role of meaning in gastric cancer patients: Relationships among meaning structures, coping, and psychological well-being. Anxiety Stress Coping.

[33] Krok D., Zarzycka B. (2020). Risk perception of COVID-19, meaning-based resources and psychological well-being amongst healthcare personnel: The mediating role of coping. J. Clin. Med..

[34] Yeung N.C.Y., Huang B., Lau C.Y.K., Lau J.T.F. (2022). Finding the silver linings in the COVID-19 pandemic: Psychosocial correlates of adversarial growth among Filipina domestic helpers in Hong Kong. Psychol. Trauma Theory Res. Pract. Policy.

[35] Yeung N.C., Tang J.L., Hui K.H., Lau S.T., Cheung A.W., Wong E.L. (2024). “The light after the storm”: Psychosocial correlates of adversarial growth among nurses in Hong Kong amid the fifth wave of the COVID-19 pandemic. Psychol. Trauma Theory Res. Pract. Policy.

[36] Boyatzis R.E. (2006). Transforming Qualitative Information: Thematic Analysis and Code Development.

[37] Fredrickson B.L. (2006). Unpacking positive emotions: Investigating the seeds of human flourishing. J. Posit. Psychol..

[38] Epel E.S., McEwen B.S., Ickovics J.R. (1998). Embodying psychological thriving: Physical thriving in response to stress. J. Soc. Issues.

[39] Zautra A.J., Reich J.W., Guarnaccia C.A. (1990). Some everyday life consequences of disability and bereavement for older adults. J. Personal. Soc. Psychol..

[40] Fredrickson B.L. (1998). What good are positive emotions?. Rev. Gen. Psychol..

[41] Fredrickson B.L. (2001). The role of positive emotions in positive psychology: The broaden-and-build theory of positive emotions. Am. Psychol..

[42] Christie K.M., Meyerowitz B.E., Giedzinska-Simons A., Gross M., Agus D.B. (2009). Predictors of affect following treatment decision-making for prostate cancer: Conversations, cognitive processing, and coping. Psycho-Oncology.

[43] Danhauer S.C., Carlson C.R., Andrykowski M.A. (2005). Positive psychosocial functioning in later life: Use of meaning-based coping strategies by nursing home residents. J. Appl. Gerontol..

[44] Harvey J.H., Chwalisz K.D., Garwood G., Orbuch T.L. (1991). Coping with sexual assault: The roles of account-making and confiding. J. Trauma. Stress.

[45] Samios C., Pakenham K.I., Sofronoff K. (2008). The nature of sense making in parenting a child with Asperger syndrome. Res. Autism Spectr. Disord..

[46] Watson D., Clark L.A., Tellegen A. (1988). Development and validation of brief measures of positive and negative affect: The PANAS scales. J. Personal. Soc. Psychol..

[47] Deng W., Gadassi Polack R., Creighton M., Kober H., Joormann J. (2021). Predicting negative and positive affect during COVID-19: A daily diary study in youths. J. Res. Adolesc..

[48] Wang Y., Jing X., Han W., Jing Y., Xu L. (2020). Positive and negative affect of university and college students during COVID-19 outbreak: A network-based survey. Int. J. Public Health.

[49] Lin D. (2018). Research on the Effect of Resilience Intervention of College Students. Master’s Thesis.

[50] Chai W., Shek D.T.L. (2024). Mental health of Hong Kong university students under COVID-19: Protective ecological factors and underlying mechanism. Appl. Res. Qual. Life.

[51] Chai W., Shek D.T.L. (2024). Mental health profiles and the related socio-demographic predictors in Hong Kong university students under the COVID-19 pandemic: A latent class analysis. Psychiatry Res..

[52] Shek D.T.L., Chai W., Li X., Dou D. (2023). Profiles and predictors of mental health of university students in Hong Kong under the COVID-19 pandemic. Front. Psychol..

[53] Shek D.T.L., Chai W., Wong T., Zhou K. (2023). Stress and depressive symptoms in university students in Hong Kong under the pandemic: Moderating effect of positive psychological attributes. Front. Psychol..

[54] Fife S.T., Gossner J.D. (2024). Deductive qualitative analysis: Evaluating, expanding, and refining theory. Int. J. Qual. Methods.

[55] Braun V., Clarke V. (2019). Reflecting on reflexive thematic analysis. Qual. Res. Sport Exerc. Health.

[56] Creswell J.W., Poth C.N. (2018). Qualitative Inquiry & Research Design.

[57] Naeem M., Ozuem W., Howell K., Ranfagni S. (2023). A step-by-step process of thematic analysis to develop a conceptual model in qualitative research. Int. J. Qual. Methods.

[58] Miles M.B., Huberman A.M. (1994). Qualitative Data Analysis: An Expanded Sourcebook.

[59] Coronavirus: Timeline. https://www.defense.gov/Spotlights/Coronavirus-DOD-Response/Timeline/.

[60] CDC Museum COVID-19 Timeline. https://www.cdc.gov/museum/timeline/covid19.html#Mid-2022.

[61] Zheng L., Liu S., Lu F. (2023). Impact of national omicron outbreak at the end of 2022 on the future outlook of COVID-19 in China. Emerg. Microbes Infect..

[62] Malterud K., Siersma V.D., Guassora A.D. (2016). Sample size in qualitative interview studies: Guided by information power. Qual. Health Res..

[63] Shek D.T.L., Dou D., Zhu X., Wong T., Tan L. (2022). Need satisfaction and depressive symptoms among university students in Hong Kong during the COVID-19 pandemic: Moderating effects of positive youth development attributes. Front. Psychiatry.

[64] Lai A.Y., Lee L., Wang M., Feng Y., Lai T.T., Ho L., Lam V.S., Ip M.S., Lam T. (2020). Mental health impacts of the COVID-19 pandemic on international university students, related stressors, and coping strategies. Front. Psychiatry.

[65] Catalano R.F., Berglund M.L., Ryan J.A.M., Lonczak H.S., Hawkins J.D. (2004). Positive youth development in the United States: Research findings on evaluations of positive youth development programs. Ann. Am. Acad. Political Soc. Sci..

[66] Lai A.Y.K., Cheung G.O.C., Choi A.C.M., Wang M.-P., Chan P.S.L., Lam A.H.Y., Lo E.W.S., Lin C.-C., Lam T.-H. (2022). Mental health, support system, and perceived usefulness of support in university students in Hong Kong amidst COVID-19 pandemic: A mixed-method survey. Int. J. Environ. Res. Public Health.

[67] Braun V., Clarke V. (2006). Using thematic analysis in psychology. Qual. Res. Psychol..

[68] Kidder L.H., Fine M., Mark M.M., Shotland L. (1987). Qualitative and quantitative methods: When stories converge. New Directions in Program Evaluation.

[69] Braun V., Clarke V., Terry G., Hayfield N., Liamputtong P. (2018). Thematic analysis. Handbook of Research Methods in Health and Social Sciences.

[70] Gilgun J.F., Bryant A., Charmaz K. (2019). Deductive qualitative analysis and grounded theory: Sensitizing concepts and hypothesis-testing. The Sage Handbook of Current Developments in Grounded Theory.

[71] Pearse N., Stacey A. (2019). An illustration of deductive analysis in qualitative research. Proceedings of the 18th European Conference on Research Methodology for Business and Management Studies.

[72] Shek D.T.L., Tang V.M., Han X.Y. (2005). Evaluation of evaluation studies using qualitative research methods in the social work literature (1990–2003): Evidence that constitutes a wake-up call. Res. Soc. Work Pract..

[73] Levitt H.M., Motulsky S.L., Wertz F.J., Morrow S.L., Ponterotto J.G. (2017). Recommendations for designing and reviewing qualitative research in psychology: Promoting methodological integrity. Qual. Psychol..

[74] Folkman S., Moskowitz J.T., Hewstone M., Schut H.A.W., De Wit J.B.F., Van Den Bos K., Stroebe M.S. (2007). Positive affect and meaning-focused coping during significant psychological stress. The Scope of Social Psychology: Theory and Applications.

[75] Aldwin C.M., Revenson T.A. (1987). Does coping help? A reexamination of the relation between coping and mental health. J. Personal. Soc. Psychol..

[76] Weinstock M.W., Moyer S., Jallo N., Rider A., Kinser P. (2024). Perinatal meaning-making and meaning-focused coping in the COVID-19 pandemic. J. Reprod. Infant Psychol..

[77] Lancastle D., Boivin J. (2008). A feasibility study of a brief coping intervention (PRCI) for the waiting period before a pregnancy test during fertility treatment. Hum. Reprod..

[78] Finkelstein-Fox L., Park C.L., Kalichman S.C. (2020). Health benefits of positive reappraisal coping among people living with HIV/AIDS: A systematic review. Health Psychol. Rev..

[79] Taylor R., Thomas-Gregory A., Hofmeyer A. (2020). Teaching empathy and resilience to undergraduate nursing students: A call to action in the context of COVID-19. Nurse Educ. Today.

[80] Sun N., Wei L., Wang H., Wang X., Gao M., Hu X., Shi S. (2021). Qualitative study of the psychological experience of COVID-19 patients during hospitalization. J. Affect. Disord..

[81] Diponegoro A.M., Hanurawan F. (2022). Creativity, curiosity, open mindedness, love of learning, and perspective character strengths in students’ wellbeing during the COVID-19 pandemic. KnE Soc. Sci..

[82] Van Stee E.G. (2023). Privileged dependence, precarious autonomy: Parent/young adult relationships through the lens of COVID-19. J Marriage Fam..

[83] Shah M., Rizzo S., Percy-Smith B., Monchuk L., Lorusso E., Tay C., Day L. (2021). Growing up under COVID-19: Young people’s agency in family dynamics. Front. Sociol..

[84] Asadullah N. (2020). COVID-19, Schooling and Learning.

[85] Online Learning and Education for all During and After Covid-19 Pandemic. Financial Express. 13 July 2020. https://www.financialexpress.com/jobs-career/education-online-learning-and-education-for-all-during-and-after-covid-19-pandemic-2021940/.

[86] Navarro J.L., Brown M., Jensen T., Weinstein M., Jensen M. (2024). It isn’t just mom: Gendered provision of family and home responsibilities among emerging adults during COVID-19. Front. Psychiatry.

[87] Landi G., Pakenham K.I., Cattivelli R., Grandi S., Tossani E. (2022). Caregiving responsibilities and mental health outcomes in young adult carers during the COVID-19 pandemic: A longitudinal study. Int. J. Environ. Res. Public Health.

[88] Al Mamun F., Hosen I., Misti J.M., Kaggwa M.M., Mamun M.A. (2021). Mental disorders of Bangladeshi students during the COVID-19 pandemic: A systematic review. Psychol. Res. Behav. Manag..

[89] Alam M.K., Ali F.B., Banik R., Yasmin S., Salma N. (2022). Assessing the mental health condition of home-confined university level students of Bangladesh due to the COVID-19 pandemic. J. Public Health.

[90] Xiang M.-Q., Tan X.-M., Sun J., Yang H.-Y., Zhao X.-P., Liu L., Hou X.-H., Hu M. (2020). Relationship of physical activity with anxiety and depression symptoms in Chinese college students during the COVID-19 outbreak. Front. Psychol..

[91] Alvis L.M., Douglas R.D., Shook N.J., Oosterhoff B. (2023). Associations between adolescents’ prosocial experiences and mental health during the COVID-19 pandemic. Curr. Psychol..

[92] De Leeuw R.N.H., Van Woudenberg T.J., Green K.H., Sweijen S.W., Van De Groep S., Kleemans M., Tamboer S.L., Crone E.A., Buijzen M. (2023). Moral beauty during the COVID-19 pandemic: Prosocial behavior among adolescents and the inspiring role of the media. Commun. Res..

[93] Yang X., He Y., Luo B., Zhao L., Huang C., Liao S. (2023). Associations between adolescents’ empathy and prosocial attributes before and during the COVID-19 pandemic. BMC Pediatr..

[94] Van De Groep S., Zanolie K., Green K.H., Sweijen S.W., Crone E.A. (2020). A daily diary study on adolescents’ mood, empathy, and prosocial behavior during the COVID-19 pandemic. PLoS ONE.

[95] Haller E., Lubenko J., Presti G., Squatrito V., Constantinou M., Nicolaou C., Papacostas S., Aydın G., Chong Y.Y., Chien W.T. (2022). To help or not to help? Prosocial Behavior, its association with well-being, and predictors of prosocial behavior during the coronavirus disease pandemic. Front. Psychol..

[96] Terrier C., Chen D.L., Sutter M. (2021). COVID-19 within families amplifies the prosociality gap between adolescents of high and low socioeconomic status. Proc. Natl. Acad. Sci. USA.

[97] Gan Y., Guo M., Tong J. (2013). Scale development of meaning-focused coping. J. Loss Trauma.

[98] Ooi L., Paul E., Burton A., Fancourt D., McKinlay A.R. (2023). A qualitative study of positive psychological experiences and helpful coping behaviours among young people and older adults in the UK during the COVID-19 pandemic. PLoS ONE.

[99] Fioretti C., Palladino B.E., Nocentini A., Menesini E. (2020). Positive and negative experiences of living in COVID-19 pandemic: Analysis of Italian adolescents’ narratives. Front. Psychol..

[100] Kim J.J., Munroe M., Feng Z., Morris S., Al-Refae M., Antonacci R., Ferrari M. (2021). Personal growth and well-being in the time of COVID: An exploratory mixed-methods analysis. Front. Psychol..

[101] Perasso G., Serantoni G., Lillo C., Paoletti P., Maculan A., Vianello F., Di Giuseppe T. (2023). Resilience predictors in the post-pandemic era: A study on Italian adolescents. Ric. Psicol..

[102] Yao Y., Yao J., Chen S., Zhang X., Meng H., Li Y., Lu L. (2023). Psychological capital and self-acceptance modified the association of depressive tendency with self-rated health of college students in China during the COVID-19 pandemic. Behav. Sci..

[103] Brites R., Brandão T., Hipólito J., Ros A., Nunes O. (2024). Emotion regulation, resilience, and mental health: A mediation study with university students in the pandemic context. Psychol. Sch..

[104] Carey R.L., Bailey M.J., Polanco C.I. (2023). How the COVID-19 pandemic shaped adolescents’ future orientations: Insights from a global scoping review. Curr. Opin. Psychol..

[105] Asmundson G.J.G., Paluszek M.M., Taylor S. (2021). Real versus illusory personal growth in response to COVID-19 pandemic stressors. J. Anxiety Disord..

[106] Hannes C., Schiffer S., Von Nitzsch R. (2024). Changes in value priorities due to the COVID-19 pandemic—A 4-year cross-sectional study with German students. PLoS ONE.

[107] Henkens J.H.D., Visser K., Finkenauer C., Stevens G.W.J.M. (2022). ‘I think it’ll all blow over in the end’: How young people perceive the impact of COVID-19 on their future orientations. Young.

[108] Crane M.F., Searle B.J., Kangas M., Nwiran Y. (2019). How resilience is strengthened by exposure to stressors: The systematic self-reflection model of resilience strengthening. Anxiety Stress Coping.

[109] Falon S.L., Kangas M., Crane M.F. (2021). The coping insights involved in strengthening resilience: The self-reflection and coping insight framework. Anxiety Stress Coping.

[110] Ku Y.-R., Stager C. (2022). Rethinking the multidimensionality of growth mindset amid the COVID-19 pandemic: A systematic review and framework proposal. Front. Psychol..

[111] Ang W.H.D., Shorey S., Lopez V., Chew H.S.J., Lau Y. (2022). Generation Z undergraduate students’ resilience during the COVID-19 pandemic: A qualitative study. Curr. Psychol..

[112] Park Y., Kim I.H., Jeong Y.W. (2024). Resilience experienced by university students during the COVID-19 pandemic: A qualitative exploration based on focus-group interviews. Heliyon.

[113] Ganotice F.A., Downing K., Chan B., Yip L.W. (2022). Motivation, goals for study abroad and adaptation of Mainland Chinese students in Hong Kong. Educ. Stud..

[114] Pan J.-Y. (2011). A resilience-based and meaning-oriented model of acculturation: A sample of Mainland Chinese postgraduate students in Hong Kong. Int. J. Intercult. Relat..

[115] Ge J.L., Feldman D.B., Shu T.-M. (2023). The relationships of hope, optimism, and academic motivation with GPA among university students in Hong Kong. Psychol. Rep..

[116] Javier-Aliaga D.J., Quispe G., Quinteros-Zuñiga D., Adriano-Rengifo C.E., White M. (2022). Hope and resilience related to fear of COVID-19 in young people. Int. J. Environ. Res. Public Health.

[117] Sun Y., Lam C.B., Chung K.K.H. (2022). Being hopeful and mindful during adversity: A longitudinal study on college students’ adjustment during COVID-19. Mindfulness.

[118] Gander F., Wagner L. (2022). Character growth following collective life events: A study on perceived and measured changes in character strengths during the first wave of the COVID-19 pandemic. Eur. J. Pers..

[119] Shek D.T.L. (2021). COVID-19 pandemic and developmental outcomes in adolescents and young adults: In search of the missing links. J. Adolesc. Health.

[120] Gelles L.A., Lord S.M., Hoople G.D., Chen D.A., Mejia J.A. (2020). Compassionate flexibility and self-discipline: Student adaptation to emergency remote teaching in an integrated engineering energy course during COVID-19. Educ. Sci..

[121] Yu Z. (2022). Sustaining student roles, digital literacy, learning achievements, and motivation in online learning environments during the COVID-19 pandemic. Sustainability.

[122] Stavridou A., Stergiopoulou A., Panagouli E., Mesiris G., Thirios A., Mougiakos T., Troupis T., Psaltopoulou T., Tsolia M., Sergentanis T.N. (2020). Psychosocial consequences of COVID-19 in children, adolescents and young adults: A systematic review. Psychiatry Clin. Neurosci..

[123] Dotson M.P., Castro E.M., Magid N.T., Hoyt L.T., Suleiman A.B., Cohen A.K. (2022). “Emotional distancing”: Change and strain in U.S. young adult college students’ relationships during COVID-19. Emerg. Adulthood.

[124] Brammer M.S. (2020). Student Resilience and COVID-19. SSRN.

[125] Herbers J.E., Hayes K.R., Cutuli J.J. (2021). Adaptive systems for student resilience in the context of COVID-19. Sch. Psychol..

[126] İme Y., Ümmet D. (2022). Adaptation of emotional flexibility scale: Its association with subjective well being and resilience during Covid-19 pandemic. Child Indic. Res..

[127] Pretorius T.B., Padmanabhanunni A. (2023). Toward a positive life beyond COVID-19: Problem-solving appraisal as a resistance resource in the relationship between stress and well-being in students. Healthcare.

[128] Frydenberg E. (2017). Coping and the Challenge of Resilience.

[129] Shek D.T.L. (2006). Adolescent developmental issues in Hong Kong: Relevance to positive youth development programs in Hong Kong. Int. J. Adolesc. Med. Health.

[130] Shek D.T.L. (2006). Conceptual framework underlying the development of a positive youth development program in Hong Kong. Int. J. Adolesc. Med. Health.

[131] Lincoln Y.S., Guba E.G. (1985). Naturalistic Inquiry.

[132] Hays D.G., McKibben W.B. (2021). Promoting rigorous research: Generalizability and qualitative research. J. Couns. Dev..

[133] Lai F.H.Y., Siu A.M.H., Shek D.T.L. (2015). Individual and social predictors of prosocial behavior among Chinese adolescents in Hong Kong. Front. Pediatr..

