# Supplementary material for: Meaning-Focused Coping in University Students in Hong Kong During the COVID-19 Pandemic: A Qualitative Study
Source: Int J Environ Res Public Health. 2025 Apr 15;22(4):614. doi: 10.3390/ijerph22040614 (PMC12027191; doi:10.3390/ijerph22040614)
Supplement: Supplementary file 1 [file ijerph-22-00614-s001.zip › ijerph-3503566-supplementary.pdf]

Supplemental Table S1. *Additional illustrative quotes*

Meaning-Focused Coping and Perceived Benefits	Sub-themes	Quotes
**Realigning priorities**	Cherished the present and planned for the future	*For the lesson learned, I did learn how fragile life is. Although none of my family and friends passed away because of the virus, I have friends who have close ones passed away because of the virus. Besides, I saw on the news people died because of the pandemic every day. I think if we didn't have the pandemic, our lives could be better. At least our close ones could stay with us longer. So, I think that life is very fragile indeed. We should not struggle with things that happened in the past within our limited time. As I just said, the things that I thought were very serious before, I don't think that it is so serious after experiencing the pandemic. Things would not be so serious that they would limit your chance of survival. I have learned to take things lightly, not get too entangled in anything, and just live in the present. Living a life that makes me feel meaningful and happy, which I think is good enough.* (Mainland Chinese student, group C, M-C-4).
	Had more self-reflection, leading to personal growth and future planning	*I think I have more time to think, especially because I can't go out and I can only stay at home, and a lot of activities have been canceled, so I actually have many free time, or from another point of view, more decadent time. At the initial stage of the pandemic, I had more thinking about different aspects of life: if this situation continues, what would be the subsequent development? At that time, I was not doing so well in many aspects of my life, especially I might have been slacking off in my studies. If this situation continues, would I be in a very bad situation? I thought a lot about these issues.* (Student who coped well, group A, C-A-5).
**Adaptive goal processes**	Had a more positive attitude towards life, including being proactive and	*For the lessons I have learned in the pandemic, it's the importance of enjoying life. I used to worry a lot about the future. When I was in high school and even younger, I was very fond of delayed gratification. I liked to finish every task as soon as possible, so I could harvest in the future. It is similar to what the previous student said. I wanted to study hard, then have fun, travel the world, and do whatever I want after graduating from high school. However, it was not the reality at the time. The reality was "everything is constantly changing". Something that is possible and meaningful to do now, might become impossible to implement in a month, or even a week later, or it might become meaningless. So, for every opportunity now, no matter whether it is an entertainment or becoming an*

	independent, gaining more self-control, and enjoying life	*exchange student, we should seize it. Because the future is too unpredictable, so we have to seize every opportunity.* (Mainland Chinese student, group A, M-A-2).
		What I have learned is to take advantage of every opportunity. I used to think that I will have a lot of time in four years [of university study]. However, after a semester, I realized that if I had not learned anything, the time had already gone by. So, if you do not pay attention in class, you might miss many things. If you ask your teachers individually, the teachers might miss your email, or they may have already forgotten what they said during the lecture. So, the teachers may need time to think, then they may tell you an answer different from the one taught in the lecture. (Student with financial difficulty, group B, F-B-7).
**Benefit finding**	Were more accepting, flexible, and open-minded and worried less	*I think we should be more open-minded. Because of this pandemic, we cannot control a lot of things. Many of the time, the external environment changes quickly. Maybe we have this situation today, but we may have another situation tomorrow. So, firstly, we must give more time for ourselves to adapt to this environment. Secondly, we must be more open-minded and less obsessive. For some issues, maybe we can't do it [in this way]; if we try another method, maybe we could do it. For example, having lessons during the pandemic was not feasible, then we could do online classes. And if we cannot do online classes, we could try other ways, for example, recording the lessons and watching them later. I think we must be more open-minded, and not be too persistent to use old methods to achieve goals or objectives.* (Student with financial difficulty, group C, F-C-9).
	Increased sense of responsibility and self-perceived personal growth	*I feel that my thinking is more mature than before. In the past, when I didn't have part-time jobs, I felt that spending money was nothing and I didn't know how hard it is to earn money. I started working for some part-time jobs after entering university and before the pandemic. However, those part-time jobs have stopped because of the pandemic. My family members need to make money, and they have worked really hard, not to mention those sudden changes, such as business closing for no reason. So, I have realized that it is not easy to work and make money, and I believe that I would contribute more to my family when I work in the future.* (Student with financial difficulty, group B, F-B-2).
	Cherished relationships with family members and friends	*I found that everyone, including our families, friends, and myself, have faced various difficulties during this three-year pandemic. Even though wearing face masks has increased our physical distance from others, I have come to appreciate the importance of having support from others. I am grateful to have a group of friends who have been always supportive of me during these challenging times. Even during the toughest moments, we would find places to hang out or engage in activities together, which made the difficult times more bearable. This experience has made me value my relationships with others more and I look forward to the time when we can take off our masks and breathe fresh air without worries.* (Student who coped well, group B, C-B-3).

		As I have seen many old people died from the disease, I want to spend more time with my grandparents. Since my grandparents are not living with me, they would do COVID-19 antigen tests more frequently. If the results are negative, I would visit my grandparents. […So, do you think you are closer to them and have spent more time with them during the pandemic?] Yes, because I lived far away for school, it could take more than one hour for me to get to their house, so it was very difficult for me to visit them. (Student with financial difficulty, group B, F-B-6).
	Were more open to communicating with strangers and showing care to society	*I pay more attention to the news or social issues now. I try to connect myself to the things happening in the society. [Before the pandemic], I thought that these things were very far away from me. However, because of the pandemic, I realize that these things might happen to me very quickly, so I would pay attention to the news more often now.* (Mainland Chinese student, group B, M-B-9).
**Benefit reminding**	Maintained hope	*Maintaining hope is crucial. You have to hold onto the belief that one day things will return to normal. However, it depends on whether you can wait long enough to see it happen.* (Student who coped well, group B, C-B-6).
**Infusing ordinary events with meaning**	Accepted the self and dealt with negative emotions	*Because of the pandemic, we often use the Zoom [for online meetings] or wear masks. I used to be a bit self-abased and would avoid eye contact with others on the street. However, somehow I feel brave after wearing a mask, and I would look at others and I feel that I have made progress. I think when I take the mask off, I would not be the same as before and I will be a little more confident.* (Student with financial difficulty, group A, F-A-2).
	Learned to deal with loneliness	*In terms of personal growth, I think I have improved. I have also learned how to enjoy time alone. In the past, I was very afraid of being alone. However, during the pandemic, I have to spend a lot of time alone. Rather than being afraid of being alone, I have learned to find ways to enjoy being alone.* (Student who coped well, group C, C-C-2).
**Perceived benefits**	Enhanced adaptability and flexibility	*For adaptability, I did not have the experience of having fully online lessons, and I experienced them last semester. I was able to adapt to this kind of learning mode, and to communicate with classmates and teachers under this mode.* (Mainland Chinese student, group B, M-B-9).

	Enhanced independence	*Furthermore, my independent thinking ability has improved. During the pandemic, there has been a lot of true and false information coming out. I remind myself constantly that we should have independent thinking to determine the authenticity of the information. So, my ability in this aspect has improved.* (Student who coped well, group C, C-C-6).
	Strengthened problem-solving skills	*I feel that I have developed the ability to solve difficult problems. For example, when I was in the student-exchange program, the "Circuit Breaker" measure was implemented in Hong Kong. Fortunately, this measure was relaxed soon after, and flights from a few countries, including Canada, were allowed to land in Hong Kong. I was trying to return to Hong Kong from Canada at the time. When they just launched this policy, I planned to fly to Singapore first. The flight arrangement at that time was very chaotic. But I realized I might be exposed to the COVID-19 virus upon arrival in Singapore. I had to find a better solution, so I chose to fly back to Hong Kong directly from Canada.* (Student who coped well, group B, C-B-2).
	Strengthened self-discipline	*I think my discipline and time management have improved. During the pandemic, most of the time is left to oneself. However, sometimes I felt that I have so much time and I would procrastinate. I thought that I have a lot of time, so I could do certain things tomorrow or after having a meal. In the end, I was not able to do anything. During the pandemic, there was a moment when I stopped and reflected on how I had used the time. I found that I used most of the time to do things that were meaningless, such as scrolling my phone all day long without gaining anything at all. On the contrary, it made me felt even more tired mentally. Then I went online to see how other people manage their time and control themselves. Some activities can bring you short-term happiness, such as playing games. It is fun to play them. For studying, it is painful when we do it, but we will have a reward afterward. Then, I knew how to control myself. I would put more time into activities that could bring long-term rewards, instead of activities that would bring no benefit to me, or only short-term happiness.* (Student who coped well, group C, C-C-2).
	Learned to seek help and use online platforms	*However, I think there's another aspect that is even more important. When you have a problem, don't hesitate to seek help or ask questions just because you have limited opportunities to meet your classmates or teachers. Regardless of the circumstances, you should strive to resolve your issues and never let shyness prevent you from seeking clarification or assistance. There are always resources around you that you can utilize, whether you choose to use them or not depends on your willingness to ask for help.* (Student who coped well, group B, C-B-6).
	Increased hygiene awareness	*I think I have definitely experienced personal growth. It is because I was born after 2000, and when I was still a kid, I thought about what would happen if SARS happens again. Little did I know we will experience the COVID-19 pandemic one day. When a hypothesis becomes a reality, it has a positive*

		impact on my cognitive and psychological growth. During the SARS outbreak, everyone gained some basic health knowledge. Now, with COVID, everyone's knowledge of the pandemic has been consolidated. For people of my age, we have more and deeper understanding. First, my hygiene awareness has improved a lot. Before the pandemic, my daily habits were more casual, such as rubbing my eyes and nose, which I would do sometimes. However, during the pandemic, we wear masks, so we could avoid this kind of behavior. (Student who coped well, group C, C-C-1).
	Improved relationships with family members and friends	*The restrictions under the COVID-19 pandemic have limited our opportunities for going out and having fun. Consequently, we have less time for recreational activities, but more time for chatting with friends. Instead of meeting up in-person every time, we communicated through platforms like Signal or WhatsApp, and these conversations tended to become deeper as they continued. That's been my experience. So, in a way, I have more communication with friends.* (Student who coped well, group A, C-A-6).
